# Atomically dispersed high-loading metals and metalloids on a graphene quantum dot-support for selective electrochemical desulfurization

**DOI:** 10.1039/d5ra06256j

**Published:** 2026-01-06

**Authors:** Zahra Mohammadi, Zarrin Es'haghi, Ali Ahmadpour, Hongyang Liu

**Affiliations:** a Department of Chemistry, Payame Noor University (PNU) 19395-4697 Tehran I. R. of Iran zeshaghi@pnu.ac.ir; b Department of Chemical Engineering, Faculty of Engineering, Ferdowsi University of Mashhad Mashhad Iran; c Shenyang National Laboratory for Materials Science, Institute of Metal Research, Chinese Academy of Sciences Shenyang 110016 P. R. China

## Abstract

The amendment of electronic structures in heterogeneous catalysts has mainly been established as one of the most efficient approaches for enhancing catalytic performances in redox reactions. Herein, nitrogen-doped graphene quantum dot nanosheets with atomically dispersed metal-based nanocomposites were fabricated through a facile route and designed for the oxidative desulfurization of dibenzothiophene (DBT). The electron-enriched structure of metal-based nanocomposites is endowed with remarkable intrinsic oxidative desulfurization activities. The catalysts were characterized by FTIR, X-ray powder diffraction (XRD), field emission scanning electron microscopes (FESEMs), energy-dispersive spectroscopy (EDS), Raman spectroscopy and photoluminescence spectroscopy. This study focuses on the role of atomically dispersed metals in desulfurization treatments. As talented candidates, metal-N-GQD catalysts with metal atoms present as atomically dispersed metals (Au, Ag, Rb, Se, and Cu) were investigated as model catalysts. Based on the data obtained, the most quenching result refers to gold, which suggests that Au occupies almost all the available sites on the catalytic surface. The results established that the noncatalytic process has a greater execution in the high-impact oxidation of dibenzothiophene (DBT). This indicates that the mechanism of DBT adsorption on modified GQDs is affected by both the surface chemistry and pore structure of the adsorbents.

## Abstract 2

The amendment of electronic structures in heterogeneous catalysts has mainly been established as one of the most efficient approaches for enhancing catalytic performances in redox reactions.

## Graphical abstract

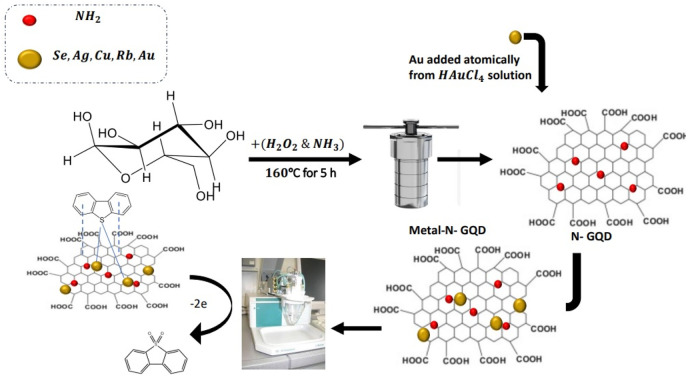

## 1. Introduction

The quality of crude oil affects the quality of the refined products in the oil refining process. Obviously, the use of cleaner crude oil containing less sulfur and fewer heavy metals averts pollution. Sulfur compounds in crude oil are one of the most significant sources of environmental contamination on earth.^1,2^ Sulfur is an integral component of crude oil present in gasoline and diesel unless it is removed somehow. In oil refining, those parts that mainly consist of nonaromatic sulfur compounds with a low boiling point are easily removed from the fuel. However, sulfur compounds, such as compounds with thiophene rings, which have a higher boiling point, are more difficult to remove than mercaptans and sulfide compounds.^3^

Likewise, the sulfur in the fuel of vehicles leads to the release of harmful sulfur-containing gases like SO_2_ and SO_3_, which are precursors to acid rain and are harmful to the environment. Therefore, extraction and refining methods must be able to eliminate sulfur from rough oils.^4^ There are various strategies to achieve fuel with safe sulfur content, for example, diesel sulfur content below 10 mg L^−1^ or 15 mg L^−1^ (the maximum permitted sulfur content in Europe and the United States).^5^

Various methods, such as hydrotreatment, also called hydrodesulfurization (HDS),^6^ oxidative desulfurization (ODS),^7^ adsorptive desulfurization^8,9^ and extractive desulfurization,^10,11^ have been developed to remove sulfur compounds.

The efficacy of adsorptive desulfurization is typically dependent on textural properties. Their advantageous characteristics include high pore volume, wide contact surface, selectivity, effective surface-active sites, and good stability.^12^ Microporous materials, such as zeolite and bentonite; mesoporous materials, such as activated carbons, metal–organic frameworks, and metal oxides; and nano-porous adsorbents, such as functionalized nano-porous carbon-based adsorbents and carbon dots, have been used for the desulfurization of model and real fuels. The satisfactory results of our previous research indicated that nano-porous carbon-based materials are good nanostructure sorbents for sulfur removal.^13^

In many recent studies, nano-porous graphene has been considered the most suitable adsorbent for a large group of materials. Graphene consists of planar sheets of carbon atoms that are strongly packed into a two-dimensional (2D) honeycomb lattice. It is a fundamental building block for carbon-containing materials, along with all other dimensions. Due to the high surface area and porosity of the nano-porous graphene, its high adsorption capacity was noticed.

However, in this study, we decided to use a better adsorbent for the desulfurization process. In this regard, we searched the available scientific sources, and the investigations led to the following results. In recent studies, graphene quantum dots (GQ-dots) have been used as alternatives to conventional graphene.^14^

Graphene quantum dots (GQDs) are small fragments of graphene that exhibit quantum confinement effects due to their nanoscale dimensions. Due to their extremely small size, quantum confinement (or in other words, the spatial localization of electron–hole pairs in one or more dimensions), biocompatibility, low toxicity, stability and water solubility, they are excellent candidates for adsorption processes.^14^

GQDs with numerous active sites can self-assemble into honeycomb-like structures. These features make them operative catalysts and adsorbents in different fields. GQDs can be stronger than graphene and are more flexible in some configurations. Because of their substantial surface area and diminished size, they can provide a significant number of surface adsorption sites. No further modifications are required to give them hydrophilic properties and reactivity because they already have a large number of hydrophilic functional groups.^15^ The selectivity of an adsorbent is still the main limitation of its application. Adsorbent selectivity can be increased by adjusting their structural properties, such as specific surface area, porosity and surface functionality. From this standpoint, after preliminary studies, we decided to use the deposition of metal atoms on the graphene dot substrate.

Surface adsorption processes with these adsorbents can occur because of the adhesion of atoms to the substrate *via* physical forces or chemical bonds. In this category, the size of the supported metal nanoparticles plays a decisive role in adsorption activity, selectivity, and adsorbent stability. Reducing the size of the nano-sorbent to scales below ten nanometers or less with the addition of only a few atoms can lead to unexpected properties, and the activity of the sorbent can be significantly tuned even through the addition or subtraction of a single atom. There are two common methods for achieving a uniform distribution of metal atoms at the atomic scale on supports: (1) physical techniques and (2) wet-chemistry methods. Physical techniques, such as atomic layer deposition, have the advantage of precisely controlling the size of the metal species. However, the lack of chemical bonding between the deposited single atoms and the substrate, the low efficiency of these techniques and the high cost limit their use in the fabrication of heterogeneous substrates. In wet chemistry methods, the decisive issue is anchoring the metal parts on the support through a chemical reaction and preventing aggregation during the process. In this case, due to the few and insufficient anchor positions on the supporting structures and the limited surface species of the support, it is very important to strengthen the structure. However, establishing a strong bond with the metal support also prevents the aggregation of individual atoms on the surface of the substrate. For these reasons, careful control of factors such as metal deposition temperature on the support, pH, and loading rate is important.^16^

## 2. Experimental section

### 2.1. Materials and methods

Analytical grade reagents were used throughout the experiment to ensure high purity without the need for additional purification. Glucose, ammonia 25%, sodium carbonate, acetonitrile, and all metal salts, such as HAuCl_4_·3H_2_O, RbI, AgNO_3_, SeO_2_, and CuNO_3_·3H_2_O, were obtained from Merck (KGaA, Germany). Hydrogen peroxide (30% wt), chloroauric acid and dibenzothiophene were purchased from Sigma-Aldrich corporation (St. Louis, MO). Tetrabutylammonium perchlorate was purchased from Fluka (Buchs, Switzerland), and double-distilled water was used in all the experiments.

A dibenzothiophene solution in acetonitrile was freshly prepared. All experiments were carried out at room temperature (25 °C). The Glassy Carbon Electrode (GCE) surface was polished using emery paper and alumina powder (1, 0.3, and 0.05 µm in diameter).

### 2.2. Apparatus

The characterizations of graphene quantum dots and atomically dispersed metals on GQDs were performed using different methods, including ultraviolet-visible spectroscopy (UV-Vis), Fourier-transform infrared spectroscopy, fluorescence spectrometry, transmission electron microscopy, X-ray diffraction, and Raman spectroscopy.

UV-Vis spectra were obtained using a two-beam spectrophotometer, UV-T80 model of PG instrument company. Fourier transform infrared (FT-IR) spectra were obtained to determine the functional groups using an FT-IR 8400 spectrometer fabricated by SHIMADZU JAPAN. A complete set of fluorescence measurements was obtained using a PerkinElmer LS-55 fluorescence spectrometer with excitation and emission slits at a 10 nm bandpass. The morphology and size dispersion of the Metal-GQD were characterized by S-TEM model EM10C-100 kV, Zeiss Germany company. X-ray diffraction (XRD) measurements were performed using the Malvern PANalytical X'pert instrument for phase and crystalline analysis. Raman spectra were recorded on the UniDRON model of South Korea with a 532 nm laser.

Electrochemical measurements were carried out using a trace analyzer, MetRohm Model 797VA Computrace (Switzerland). The electrochemical cell involved a three-electrode system with a platinum wire as the counter electrode, the Ag/AgCl/KCl (3 M) as the reference electrode and a modified glassy carbon electrode (GCE) as the working electrode. (Model CHI104, 3 mm) was polished with 1, 0.3, and 0.05 µm powder of alumina and then washed successively in 0.1 M nitric acid, anhydrous alcohol, and twice-distilled water. The cleaned electrode after coating with the nano composite was dried in an oven at 60 °C.

### 2.3. Synthesis of functionalized GQDs

To have a green synthesis of GQDs, glucose was preferred as the carbon source, with some modifications.^17^ Briefly, 1.0 g of glucose was dissolved in 100 mL of deionized water. 50 mL of this solution was taken, and 2.5 mL of ammonia (25%) and 5 mL of hydrogen peroxide (30%) were added. This mixture was then transferred into a 100 mL Teflon-lined autoclave and heated at 160 °C for 5 h. The pale-yellow solution obtained was stirred for 1 h to eliminate residual ammonium and hydrogen peroxide. Finally, the GQD solution was dialyzed for one day against DI water using a dialysis membrane with a molecular weight cutoff (MWCO) of 1000 Da.

The production of free radicals as a result of the hydrogen peroxidase reaction causes the carbon atoms of glucose to bond together to form aromatic rings, which then form GQDs as the structural units of graphene.^18,19^ In this method, ammonium is introduced into the GQD structure using both radical and hydrogen bonding methods.^20–25^ This method is a suitable green method for preparing graphene dots.

Another method for GQD synthesis was based on citric acid. The details of this method are as follows: 2 g citric acid and 20 mL of ammonia were mixed into a 25 mL Teflon-lined stainless-steel autoclave and heated at 200 °C for 10 h. After cooling to room temperature, the obtained black liquid was added to 50 mL of water; then, the resulting solution was heated to 100 °C for 1 h to evaporate excess ammonia.^26^

### 2.4. Synthesis of atomically dispersed metal catalytic materials

A general method was optimized and used to disperse the five metals (Se, Au, Ag, Rb and Cu) atomically on the surface of prepared graphene quantum dot supports.^27^ First, 5 mL of the prepared graphene quantum dot was taken. Then, the pH of the carbon supports was adjusted to approximately 10 using a sodium carbonate solution (0.25 M), monitoring the pH during the addition. Second, a measured amount of metal salt solution (0.001 g mL^−1^) was diluted in 4 mL of water; subsequently, the pH of the solution was neutralized using a measured amount of 0.25 M Na_2_CO_3_. Subsequently, the pH-neutral metal salt solution was immediately introduced dropwise to the graphene quantum dot support at a moderate rate of magnetic stirring, at 100 °C; then, stirring was continued in an oil bath at 100 °C for one hour.^28^

In this study, the term “high-loading” refers to the relatively large mass percentage of metal incorporated into the N-GQD support (wt%). Importantly, although the overall loading is high, the characterization results confirm that the metals remain atomically dispersed as single-atom sites rather than forming nanoparticles.

### 2.5. Desulfurization procedures

The nano-catalytic dispersion extraction and oxidative desulfurization protocols, originally described in Section 4.7, have been moved here for clarity. In the dispersion experiment, acetonitrile was used as the extracting solvent, and isopropyl alcohol was used as the dispersing solvent (0.2 mL IPA + 2.2 mL ACN). Moreover, 50 µL nano-catalyst suspension was added. The extraction time was 80 s, followed by 10 min of centrifugation. For oxidative desulfurization, 20 mg L^−1^ DBT solution was treated with 1.0 mL of 30% H_2_O_2_ and stirred for 20 min at room temperature before analysis. Catalyst-free control experiments were performed in parallel.

## 3. Results and discussion

### 3.1. Characterizations

#### 3.1.1. GQD characterizations

**Fig. 1:**
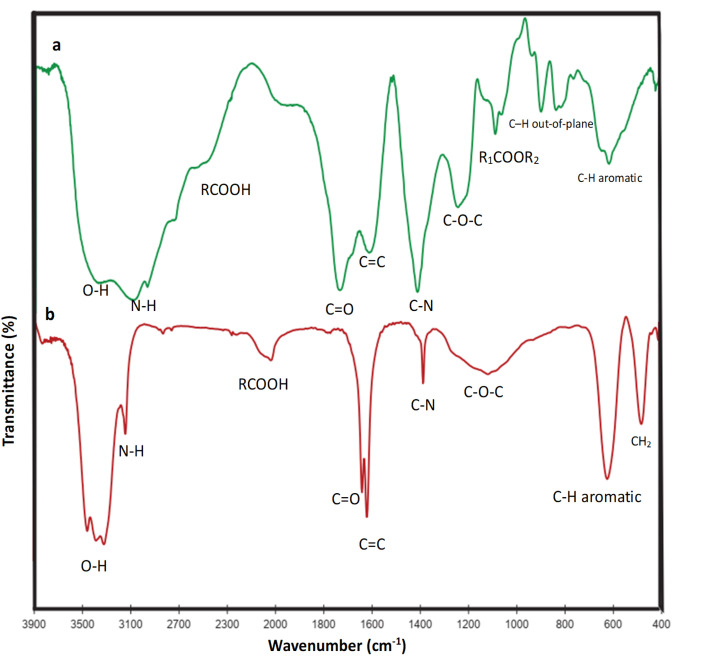
(a) FTIR spectra of the GQDs synthesized *via* the citric acid precursor method and (b) FTIR spectra of the GQDs synthesized based on glucose.

**Fig. 2:**
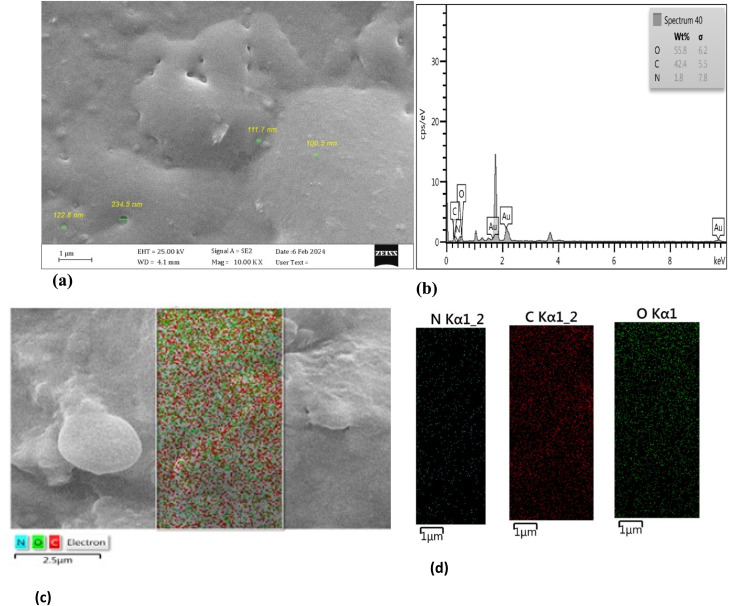
FE-SEM image and size determination of (a) the GQDs synthesized based on glucose. (b) EDS of the GQDs synthesized based on glucose. (c) Mapping from FE-SEM of GQDs synthesized based on glucose. (d) Color mapping of single elements of glucose-based GQDs.

FTIR spectroscopy was performed to investigate the existence of functional groups in the GQDs (Fig. 1). The broad peak in the region 3100–3600 cm^−1^ is related to hydroxyl groups, and two other modes at 1580 and 1650 cm^−1^ are attributed to the in-plane stretching vibrations of bonding carbon atoms with sp^2^ hybrid and COOH functional groups, respectively. Additionally, the FTIR spectra of the C=O and C–H bonds appeared at 1399 and 767 cm^−1^, respectively.^29^ The 1328 cm^−1^ bond indicates C–N bond formation in reduction with ammonia. The FTIR spectrum (Fig. 1) shows characteristic absorption bands at ∼3100–3600 cm^−1^ (O–H and/or N–H stretching), ∼1700 cm^−1^ (C=O stretching), ∼1580 cm^−1^ (aromatic C=C stretching in graphitic domains), 1328 cm^−1^ (C–N stretching, confirming N-doping), 1023 cm^−1^ (C–O stretching of ether/phenolic groups), 1399 cm^−1^ (symmetric COO^−^ stretching or C–H bending), and 767 cm^−1^ (out-of-plane aromatic C–H bending). These assignments clearly demonstrate the functional groups present in the GQDs. It should be noted that Fig. 1 shows the FTIR images, while Fig. 2 shows the SEM images.

These results confirm the synthesis of N-GQDs. The absorption peak of C=C at ∼1575 cm^−1^ confirmed the basic component of graphene.^30^ A peak at 1023 cm^−1^ (C–O bond) approved the oxidization process.^31^ All functional groups related to GQD are illustrated in Fig. 1.

In the fluorescence spectra shown in Fig. S1, GQDs from glucose and citric acid showed emission wavelengths at 400 and 515 nm, respectively, and were green in color under a handheld UV lamp. The surface state was induced by the hybrid form of the attached carbon core and edge factors, and the participant edge groups for green emission mainly originated from carboxyl and amide groups. The –OH-based hybrid structure is mainly responsible for the blue emission or probably enriches the electron density of the π structure. Therefore, at each energy level, the electron is excited, and it eventually relaxes to the surface state energy levels that determine the photoluminescence (PL) characteristics of GQDs.^32,33^

The electronic, optical and catalytic performance of GQDs depends on their size, functional groups and dopants on their surface. Larger size leads to a smaller bandgap; hence, the fluorescence emission wavelength is according to the synthesized GQDs PL wavelength. It can be concluded that the size of GQD synthesized *via* glucose as the precursor is smaller than that of GQD synthesized *via* citric acid precursor (comparing the emission wavelengths).^34^ The PL red-shift of the GQD oxygen groups, which are related to the intermediate n orbitals, leads to a decrease in the bond gap.^35^ Thus, it can be inferred that the oxygenated groups on citric acid-based GQDs are greater than on glucose-based GQDs.

There are different electron-donating groups (*e.g.*, –OH and –NH) and electron-withdrawing oxygenated functional groups (*e.g.*, –COOH and –CHO) on the surfaces of GQDs. These groups are supposed to enrich the electron density of the π-conjugated structure and cause a blueshift of the photoluminescence (PL). However, more electron-withdrawing (EW) groups, such as carboxyl, cause a PL redshift.^36^ Due to the red shift observed in the fluorescence spectra of citric acid-based GQD, it may be concluded that the carboxyl and amide groups on citric acid-based GQD are strongly anchored.

The excitation peak is located very near the absorption peak, indicating the efficient absorption and subsequent emission of N-doped GQDs (NGQDs). However, the emission intensity of glucose-based GQDs is 40 times higher than that of citric acid-based GQDs, that is, higher intensity is proportional to the smaller size of the GQDs. When the quantum dots were excited at a wavelength of 320 nm, the PL spectrum showed a strong peak at 440 nm with a Stokes shift. As the excitation wavelength increased (from 320 to 420 nm), the PL peak shifted to longer wavelengths with a rapid decrease in intensity, which is the characteristic of PL for most C-based nanoparticles.

The as-prepared NGQDs showed substantial fluorescence with a quantum efficiency of 34% (with 0.05 M quinine sulfate as a reference fluorophore), while for the undoped GQDs, the quantum yield was 2%. The UV-Vis spectra of the GQDs are presented in Fig. S2 and S3. The peak at 255 nm refers to the sp^2^-hybridized structure due to C=C and C–C, and the peak at 381 nm corresponds to the sp^3^-hybridized structure resulting from the C=O bond due to the n to π* transition corresponding to the excitation of an unpaired electron to the π* orbital. The peak at 410 nm is attributed to the formation of partially conjugated π electrons in NGQDs with high nitrogen doping. An average size of about 5 nm is enough to make an extensive partially conjugated π electron system.^37,38^ A prominent UV absorption feature peaking at ∼265 nm in GQDs is a feature of π–π* transition within sp^2^-hybridized carbon.

The morphology of the synthesized glucose-based GQD was evaluated through SEM images, EDS spectra and mapping of GQD. The results are presented in Fig. 2 and S4. GQD size may be controlled by reaction time, temperature, and reactant concentration.^39,40^ GQD syntheses, using top-down methods, usually produce GQDs with a wide range of size distributions. However, using the bottom-up method to produce GQDs, a uniform-sized particle and dispersion can be observed from the SEM images and their mapping.

Raman spectroscopy of GQD synthesized based on glucose is shown in Fig. S5a. Size and shape dependence are observed in the frequencies of both the D- and G-bands, which decrease with increasing GQD size and with decreasing defect size and density. Higher-order modes are better defined by increasing the size of GQDs and expanding their sp^2^ networks.^41^ The D-to-G band intensity ratio (*I*_D_/*I*_G_ = 1.01) indicates a relatively high proportion of defects and edge functional groups in GQDs due to their small lateral size.^42^ Further Raman information was obtained at 532 nm, where both the D and G bands were asymmetrically broadened, revealing other overlapping vibrational modes.^43^ The distinctive G band at 1604 cm^−1^ in GQDs is a manifestation of the in-plane vibrational coupling of sp^2^ carbon.^44,45^ The Raman G bond peak position exhibits a red shift upon introducing dopant atoms into the graphitic system.^46^ The redshift indicates a tensile strain in the structure, which may result from the doping of electron donating nitrogen atoms. The Raman spectra of the GQDs exhibited typical D (∼1350 cm^−1^) and G (∼1580 cm^−1^) bands, which are related to disordered sp^3^ carbon and graphitic sp^2^ domains, respectively. Although the raw spectra showed a pronounced baseline due to the fluorescence background, the baseline-corrected spectra and the calculated *I*_D_/*I*_G_ ratios are illustrated in Fig. S5b. The corrected data confirm the presence of graphitic domains and defects consistent with the FTIR results.

#### 3.1.2. Atomically dispersed metal catalyst characterizations

As can be observed from the fluorescence spectrum shown in Fig. S6, all metals cause a quenching effect on the fluorescence of graphene quantum dots, but the quenching ability of metals atomically dispersed on the substrate is not the same. They change the position of the maximum photoluminescence peak of GQD. Except for Rb, all other metals cause a significant red shift on the fluorescence peak of the graphene dot, which confirms their binding to GQD. Charge transfer occurs from metals to GQDs, resulting in a narrower band gap.^40^ This decrease in the band gap due to charge transfer is responsible for the observed red shift. Rb causes a slight blue shift, which indicates that it is not good at charge transfer and its electron donating is not as strong as others. The weaker interaction between the metal and the substrate may be a result of the rupture of an aromatic heterocycle near the rubidium atoms. The greater quenching ability could be a result of strong metal bonding with oxygen and nitrogen in the GQD. The highest quenching result corresponds to gold, which can be deduced from Au occupying almost all the available sites on the GQD surface.^47^

In addition to nitrogen dopants, N-GQDs also contain oxygenated functional groups, such as carboxyl, hydroxyl, and carbonyl moieties, which serve as anchoring sites for metal atoms. The nearly complete PL quenching observed for Au-N-GQDs indicates that Au has a particularly strong affinity to coordinate with both N and O functionalities, leading to almost full occupation of the available sites. This extensive site occupation enhances charge transfer, suppresses radiative recombination, and directly translates into superior electrochemical activity in DBT desulfurization.

For further investigation, different concentrations of copper stock solution (CuNO_3_·3H_2_O, 1000 mg L^−1^) were selected and tested, and the results related to the quenching of GQD fluorescence emission were evaluated. As can be observed from the spectrum shown in Fig. S7a, with an increase in the amount of copper, the fluorescence intensity decreases. Almost complete quenching occurred at 40 (mg L^−1^) and then remains constant with increasing quencher concentration. Two different synthesis protocols (glucose- and citric acid-derived GQDs) were employed to compare the influence of precursor chemistry on structural and optical features. However, all catalytic evaluations in this work were conducted using glucose-derived N-GQDs owing to their higher fluorescence intensity, better stability, and superior ability to anchor metal atoms. The citric acid-derived GQDs were used only for comparative purposes in optical characterization. Regarding the Cu-GQDs, the variation in the fluorescence emission spectra observed in Fig. S7 is attributed to concentration-dependent static and dynamic quenching, as well as possible aggregation effects. To clarify this point, a Stern–Volmer analysis and additional comparative spectra are now provided in SI S7c. The relatively large SVK indicates efficient quenching of GQD emissions by Cu species. A predominantly linear Stern–Volmer plot (high *R*^2^) suggests that dynamic quenching (collisional) is the main mechanism; however, the intercept (>1) suggests a possible minor static quenching contribution (complex formation). This behaviour is consistent with the partial binding/complexation of Cu to surface sites, followed by collisional interactions at higher concentrations. To gain further insight into the quenching mechanism, a Stern–Volmer analysis of the Cu-GQD photoluminescence was performed (Fig. S7c, SI). The linear plot of I0/I *versus* Cu concentration gave a good correlation (*R*^2^ = 0.965), with a Stern–Volmer constant of 9.76 × 10^3^ L mol^−1^ (0.1536 L mg^−1^). The near-linear behaviour indicates efficient and concentration-dependent quenching, which is consistent with a predominant dynamic (collisional) quenching mechanism, with a possible minor static contribution suggested by the intercept above unity.

All these procedures were performed for Rb in the same way as that for Cu. The results shown in Fig. S8a demonstrate that at a higher concentration, 0.5 mL of a 1000 mg L^−1^ rubidium stock solution, the dispersion is less than at a lower concentration, *i.e.* 0.1 mL of stock solution. The quenching decreases with increasing rubidium concentration. It can be concluded that at higher concentrations of this metal, side reactions such as complexation with OH or other reactants occur and prevent its dispersion and anchoring to the surface. At the optimal concentration of rubidium, a decrease in fluorescence intensity was observed from 823.39 to 2157. Similar studies were performed on GQDs synthesized from citric acid. The results are shown in Fig. S8b. The fluorescence emission of this GQD is lower than that of glucose-based GQD, so its use is not sufficiently efficient. Furthermore, the amount of quenching is not reasonable. This can be the result of few functional groups and double bonds that make the metal completely unable to interact with them.

**Fig. 3:**
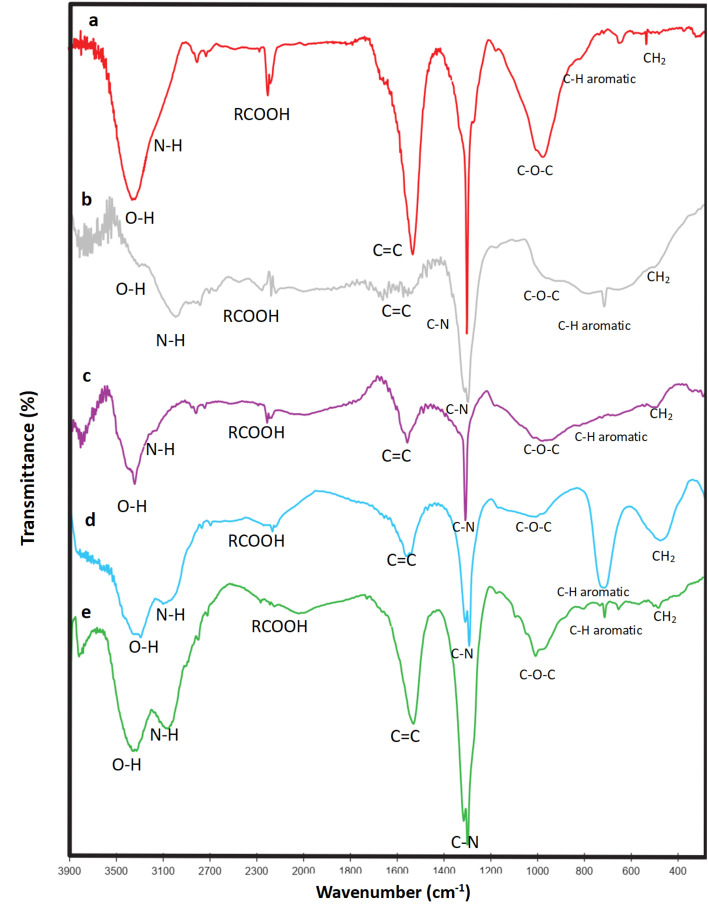
(a) FT-IR spectra of the Cu-GQDs synthesized from glucose. (b) FT-IR spectra of the Au-GQDs synthesized from glucose. (c) FT-IR spectra of the Ag-GQDs synthesized from glucose. (d) FT-IR spectra of the Se-GQDs synthesized from glucose. (e) FT-IR spectra of the Rb-GQDs synthesized from glucose.

As can be observed from the IR spectra of GQDs synthesized from glucose and citric acid precursors (Fig. 1), the intensity of all functional groups on the surface of GQD is reduced to almost negligible levels and or eliminated after atomically dispersed metals on their surfaces (Fig. 3).^48^

However, the intensity of the C–N vibration bands increases to some extent in all metal IR spectra (Fig. S9 and 3), which may be due to the low anchoring tendency of the Au atoms. However, this shows that metal atoms prefer oxygen groups instead of amines. Additionally, the absorption band's intensity is associated with the distribution of electron density in the bond; this means that increased bond polarity leads to a higher absorption band. From the XRD phase determination, we noticed that after adding HAuCl_4_, the nitrogen group accepted acidic hydrogen and converted it to NH_4_Cl, leading to an increase in bond polarity and, as a result, an increase in the intensity of the C–N peak. The dispersing of metal atoms on the surface of the GQD can be deduced from these spectra.

The C–N peak after metal anchoring became sharper, which could be a result of decreasing hydrogen bonding between N and the H connecting to oxygen groups. It can be inferred that the interaction between the metal and functional groups and defect sides is at a maximum.

The FT-IR spectroscopy of the Rb and GQDs (Fig. S9), with an optimum amount of the stock solution (0.3 mL), shows a great decrease in spectra intensity, so it is concluded that atomic dispersion is better at the optimum concentration. Additionally, the C–N stretching intensity occurring after metal dispersion is higher under these conditions compared to higher concentrations. It can be concluded that Rb bonds with carbon and oxygen functional groups and weakens their peaks because it reduces their vibrational motions.

The UV-Vis absorption spectra of the GQDs (Fig. S2) show a clear double absorption peak around 234 and 258 nm, which is attributed to the electron transfer from π → π* of the sp^2^ aromatic domain and n → π from the amino group at the edges of the GQDs, respectively. The spectra of the doped samples are characterized by a higher intensity and a broader peak.^44^ The increased intensity of the absorption feature suggests an enhancement of the π → π* transitions as a consequence of doping with N atoms, which act as electron donors.

Permatasari *et al.* reported that pyridinic-N of N-GQDs contributes a pair of electrons to sp^2^ carbon (C) atoms.^48^ The absorption tail in the range of 350–600 nm is assigned to the n → π* transition for the non-bonding electrons of O (C=O) and N (C–N).^49^

Jin *et al.* reported a reduction in the bandgap of N-GQDs with an increasing number of –NH_2_ due to the donation of its lone pair electrons to aromatic rings.^21^ In the present synthesis of N-GQDs, electron-donating –NH groups reduce their bandgap.

According to the charge transfer effect of metals and their free orbitals participating in electron exchange and resonance systems, we can observe some transformation in the GQD UV-vis spectra after introducing the metal to the GQD support (Fig. S10). The UV absorption peaks became sharper, and an obvious enhancement in intensity was observed. As the peak became sharper, we can conclude that almost all the electron donating atoms on the surface of the GQD structure have been involved in interaction and bonding to metal atoms and the electron donating of functional groups accordingly decreased. Another change is the appearance of a new peak in the visible region, which is related to n → π* electron transfer of both metal atoms and decreasing bond gap energy of GQD.

In the Se UV-vis spectra, there is no distinguished peak in the visible region. By considering its position in Mendeleev's table, it can be interpreted thoroughly. This element is in the number six of the main group and has two free paired electrons after bonding its two lone valance electrons to functional sites on the GQD surface; it seems that a stable structure is achieved, so it has no tendency to participate in its two paired valance electrons in molecular n orbitals. Compared to other metals, its UV peak is sharper, which can be a result of not participating in the conjugation system.

Silver (Ag), copper (Cu) and gold (Au) are all elements located in Group 11 of the periodic table, so they have the same electronic effect as can be observed from their spectra. Their increasing effects on the intensity and shape of the GQD peak and the appearance of a broad peak in the visible region are almost similar.

Rb is in the first main group of the table, so it has a good tendency to electron donating. As a result, it has broadened the UV spectra and increased its intensity more than the other metals.

In order to confirm the sp^2^ carbon on the GQD structure, Raman spectroscopy was performed. All the GQD samples exhibit strong dispersed fluorescent backgrounds in the wavenumber ranging from 100 to 3000 cm^−1^, centered at ≈1600 cm^−1^.^50^ The characteristic D and G bands overlay this strong fluorescent background. The Raman spectrum of graphene is dominated by two main bands: one peak at ∼1580 cm^−1^, referred to as the G band, and another peak at ∼2700 cm^−1^, identified as the 2D band (or G′ band). It also shows a D band at approximately 1350 cm^−1^. If a sample shows no evidence of G or 2D bands, it is not considered graphene. In graphene sheets, the intensity of the D band is considerably lower than that of the G band; in contrast, for GQDs, the intensity of the D band is higher than that of the G band.

**Fig. 4:**
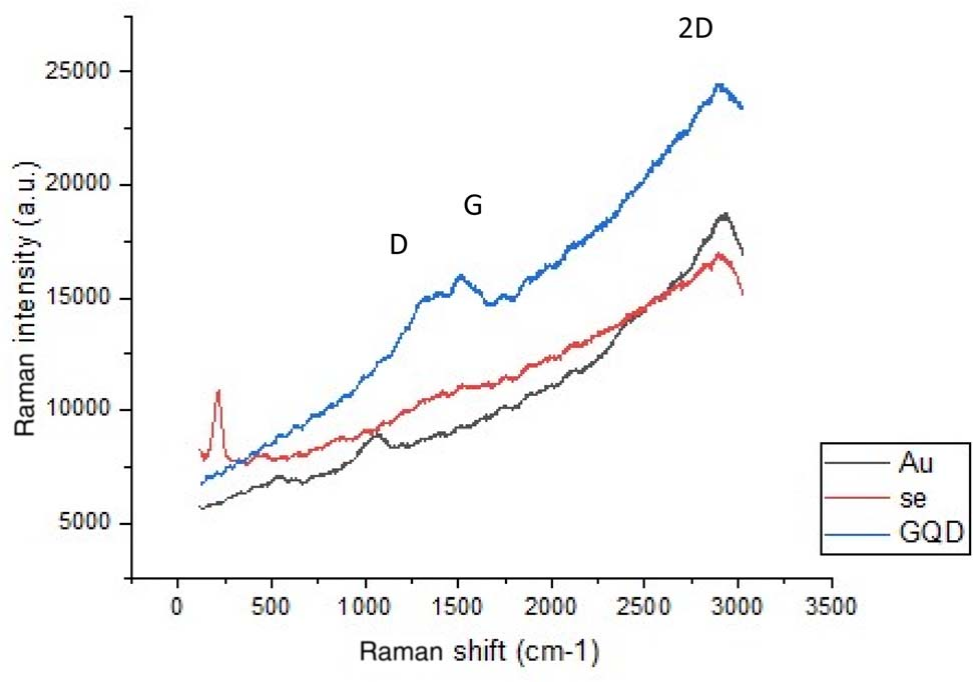
Raman spectra (plots with no smoothing and no subtracting baseline) of the GQDs, Se-GQD and Au-GQDs.

A reduction in GQD size correlates with an enhancement of the integrated intensity ratios of the D and G bands (*I*_D_/*I*_G_). From the obtained Raman spectra (Fig. 4), the GQD-characterized peaks are significant. Bond D refers to the intensity and number of defects and sp^3^-hybridized carbon on the graphene quantum dot surface. The G and 2D bands refer to the graphite structure of carbon, and the 2D band shifts to a higher wave number in comparison to graphene. This is another confirmation of the graphene quantum dot structure. The increase in the D bond is a result of oxidation and an increase in the band gap.

To investigate probable changes in Raman spectra after adding metals, Se and Au were chosen. Fig. 7 shows their Raman spectra.

As depicted in Fig. 7, after the atomic dispersion of gold on the surface of GQD, the intensity of D and G bands decreases. This could be due to reduced polarizability as a result of gold anchoring. Gold can tighten the carbons surrounding the electron cloud, thus reducing polarizability. A slight red shift is observed in the GQD Raman spectra after introducing the metals, and this red shift can be attributed to the charge transfer effect of metals that decreases the band gap energy. Basically, the red shift indicates that the frequency of quantum particles interacting with the incident photon decreases, and the blue shift indicates that it increases. The spectrum exhibits a red shift because the crystallinity of the material is enhanced. Further, Raman scattering is an inelastic phenomenon, characterized by a change in the energy (or frequency, or wavelength) of the scattered light compared to the incident light, resulting from its interaction with the material. The quantum mechanical nature of this process is demonstrated by the interaction of incident light with the vibrational modes of the material. This interaction leads to a discrete exchange of energy quanta, resulting in a change in the energy of scattered light. The spectral shift, either blue or red, quantifies the energy transfer between the incident light and the material's vibrational modes (phonons).

Here, we can explain the red shift observed in the GQD Raman spectra as follows: the metals increase the crystallinity of the structure so that a red shift occurs. The red shift of Se is greater than that of Au, and this is due to the semiconducting behavior of Se, which takes more energy from an incident photon to excite its two lone pair electrons to higher orbitals. However, Au, except for one bonding electron, does not have any other lone electron in the valance orbital, so it cannot gain the incident light energy so much; therefore, a very small red shift is observed due to the total charge transfer effect.

If metal oxide derivatives are present on the surface of the synthesized catalysts, we can obviously observe the corresponding signals, but no such peaks appear in the spectra, indicating the purity of the atomically metallic surface characteristics of the catalyst.

Although the precise wt% values of metal loading were not determined (ICP-OES analysis was not performed), the atomic dispersion of the metals on N-GQDs is strongly supported by multiple characterizations. TEM images show no observable nanoparticles or nanoclusters, EDX mapping demonstrates homogeneous distribution of the metals, and UV-Vis/Raman analyses confirm changes consistent with substitutional/atomic incorporation rather than cluster formation. Together, these results verify that the metals remain atomically dispersed under the present synthesis conditions.

#### 3.1.3. Morphological analysis

**Fig. 5:**
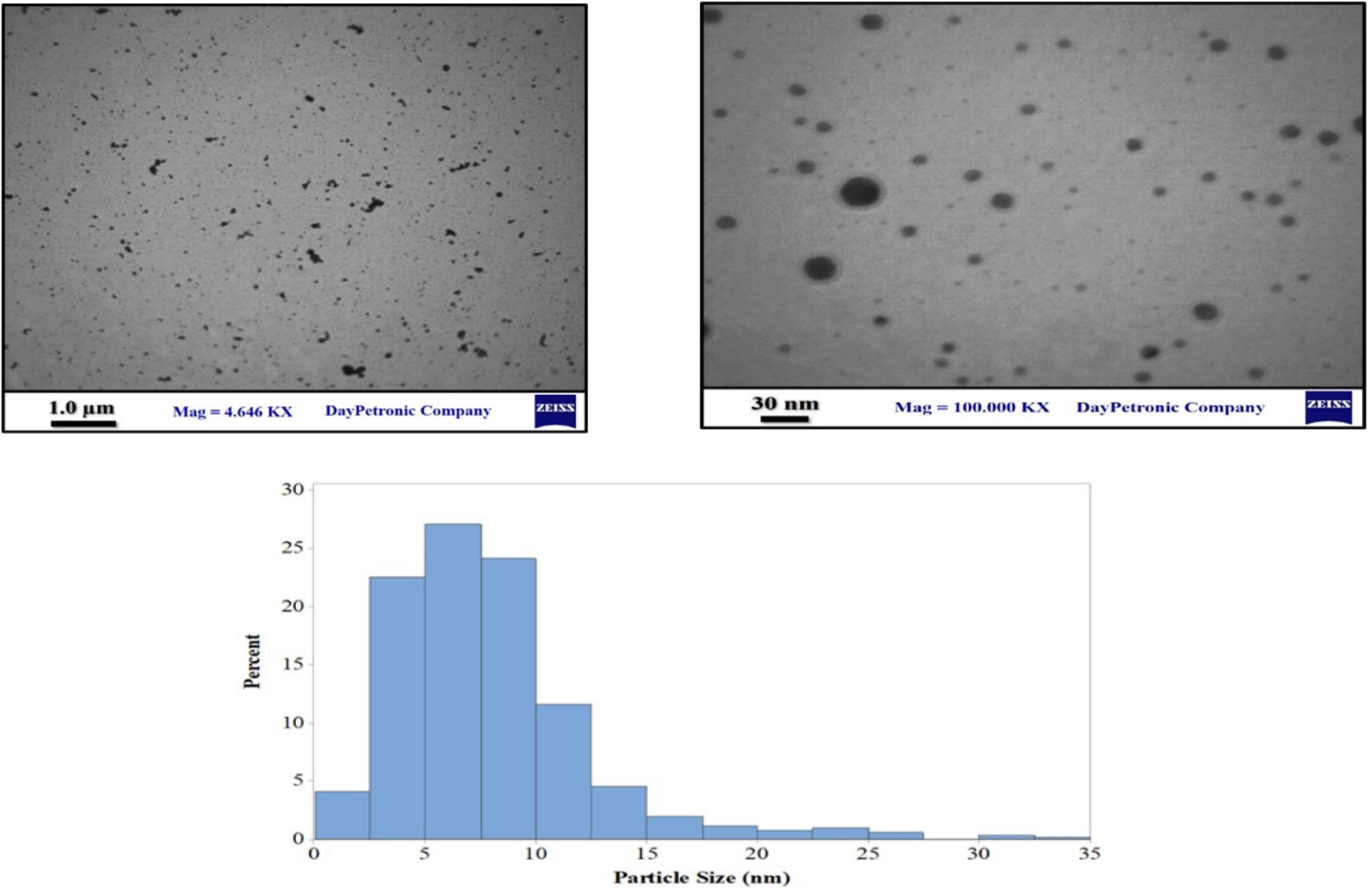
TEM micrographs and particle size distribution histograms of the Au-GQD.

Considering the STEM images depicted in Fig. 5, the spherical shape and uniform dispersion of gold on the GQD surface can be observed. The histogram shows that most of the particle sizes are below 15 nm, and the most abundant size ranges from 2 to 6 nm with spherical and cubic shapes.

The morphology and size characterizations certainly prove the successful anchoring of Au nanoparticles as single atoms with NGQDs without using any surfactant or reducing agent.

**Fig. 6:**
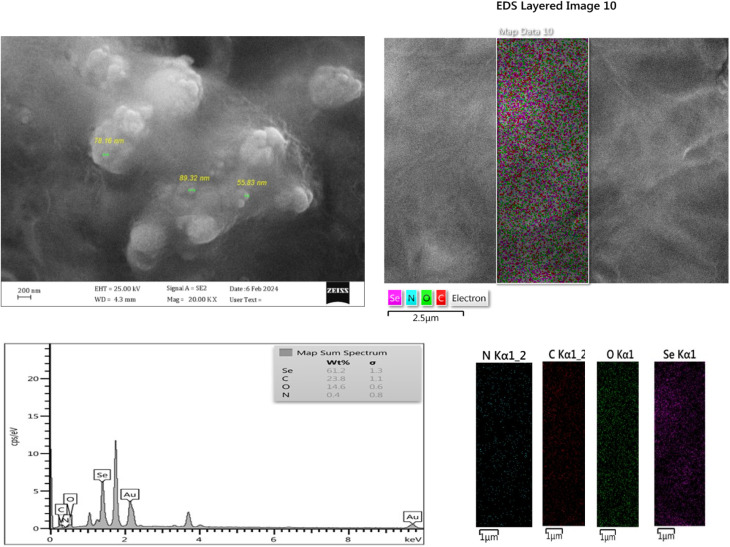
FE-SEM image of the Se-GQD, which shows semi-homogeneous dispersion of Se-GQD nanosheets, and EDS elemental mapping of the nanocomposite.

A uniform dispersion of Se is illustrated from the FESEM and EDS mapping shown in Fig. 6.

**Fig. 7:**
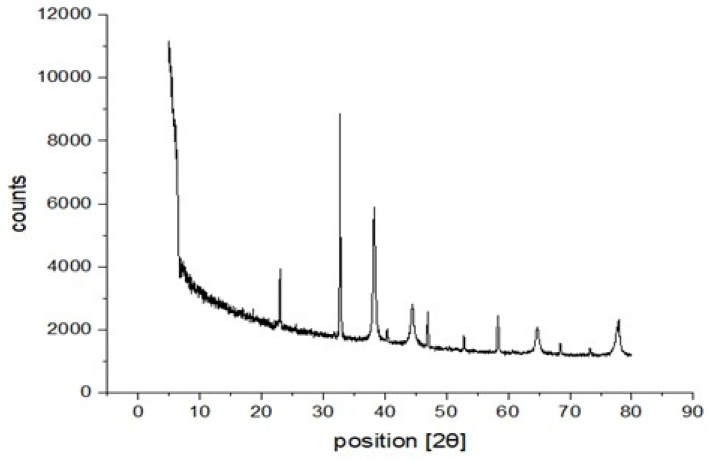
XRD results of the deposited nanostructure Au-GQDs.

The XRD results of the Au-GQD are shown in Fig. 7. The XRD peaks at positions of 23°, 40°, 46°, and 58° are related to the characteristic structure of the GQD. The peaks are broad due to the small size of the GQDs. The position of the (002) diffraction peak at ∼26.0 is due to sp^2^-hybridized carbon in the GQD's basal plane.^51^ A significantly narrower line width was observed for the Au-N-GQD peak compared to the other samples. This could be due to the reduced disorder/vacancy in the basal planes of the N-GQDs. The calculated *d* spacing ranges from 0.3857 to 0.15 nm. The observed values align with the reported data for GQDs synthesized using alternative approaches.^52^ The small difference in *d* spacing between the bulk graphite and the GQDs results from the presence of functional groups, *i.e.*, –O–H, –C–H, and –C–O–R, on the surface of the GQDs, which enlarges the spacing of the graphene layers.

The characteristic peaks corresponding to (111), (200), and (220) of Au are located at 2*θ* = 38.29°, 44.43° and 64.68°, respectively. The results indicate that the sample is composed of crystalline gold.

Another phase detected at a position of 32° is related to ammonium chloride. Here, the HAuCl_4_ salt was used as a gold source. The acidic hydrogen of this salt bonds to free unreacted ammoniac and makes ammonium chloride.

## 4. Electrochemical desulfurization

Ling *et al.* reported that the π → π stacking between the graphene and absorbed molecules could shorten the distance between the molecules and the substrate, which could facilitate efficient charge transfer from the substrate to the target molecules.^53,54^

GQDs with larger surface areas, more exposed edges, and greater numbers of dangling bonds exhibit improved adsorption capacity for target molecules. Liu *et al.* described the dangling bonds of GQDs as preferential adsorption sites for R6B molecules.^55^ Among all synthesized GQDs, the sp^2^ domain in N-GQDs acquires higher electronic charge contents, which facilitates maximum charge transfer to the target molecules by π → π interaction.

A novel graphene-like nanocomposite modified with metal atoms was prepared *via* a simple process in this research. They showed very valuable catalytic effects on desulfurization in this research. Many appreciated properties of this material are yet to be clarified and therefore are expected to stimulate a lot more experimental study.

Dibenzothiophenes (DBT), particularly 4,6-dimethyl dibenzothiophene, which is considered resistant to chemical reactions, cannot be removed by common methods. Therefore, to assess the value of this new nanoparticle for desulfurization, DBT was used as a model oil.

For the noncatalytic control experiments, both a bare glassy carbon electrode (GCE) and an electrode modified with N-GQDs without metal were tested. In both cases, the electrochemical response toward DBT oxidation was significantly weaker than that of the M/N-GQD-modified electrodes, confirming that the enhanced activity originated from atomically dispersed metal sites.

### 4.1. General processes

An optimized amount of modified adsorbent was placed on the cleaned surface of the glassy carbon electrode with the aid of a sampler and dried in the oven at 60 °C. Then, an appropriate amount of dibenzothiophene in acetonitrile was placed inside a 10 mL balloon. After that, an optimized amount of tertbutyl ammonium perchloride was added as an electrolyte and was finally filled with 10 mL of acetonitrile. This prepared solution was placed in the electrochemical cell, optimized conditions for CV and DPV analysis were applied and the current was plotted against potential.

**Scheme 1:**
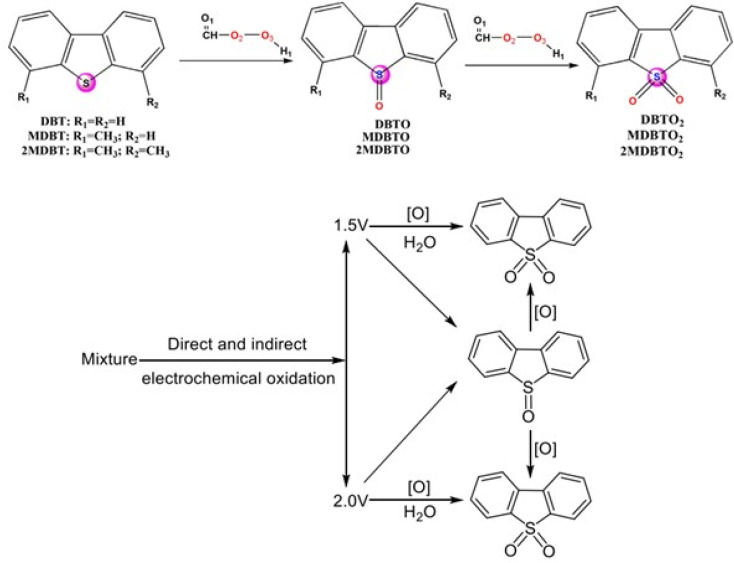
Electrochemical oxidation of DBT to yield its corresponding sulfoxide and sulfone at 1.50 and 2.00 V in acetonitrile–water (0.01 M tetra butyl ammonium chloride).

According to DFT simulations performed in previous literature,^56^ the effect of nitrogen on doping metals can be understood. N-GQD has a negative charge because nitrogen has high electronegativity and can strongly accept electrons from the metal and chemically bond the metal to the GQD surface. Dibenzothiophene can adsorb on the surface of the ADC, and after its oxidation to sulfoxide or sulfone, the compound becomes electron poor, and substitution occurs at the meta position^57,58^ (Scheme 1).

### 4.2. Optimization of electrochemical parameters

To find the optimized conditions, surface response methodology and Box–Behnken design using Minitab 16 were applied. We optimized the factors in three parts: first, synthesis of atomically dispersed metal; second, cyclic voltammetry analysis; and third, differential pulse voltammetry (DPV) analysis.

#### 4.2.1. Optimized synthesis of nano-catalyst

**Fig. 8:**
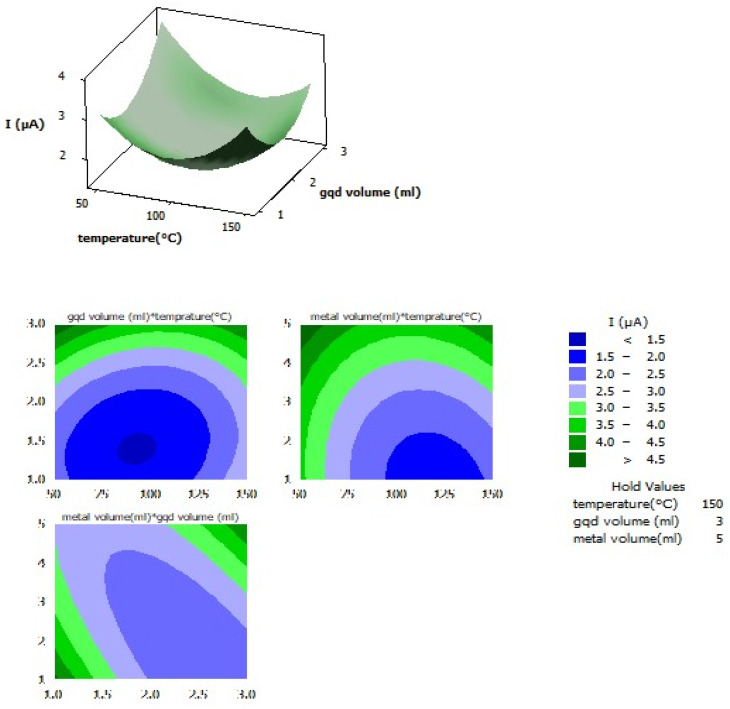
Surface and contour plots of the interaction effects of the GQD atomically dispersed Au nanocomposite as the adsorbent.

The experimental design of atomically dispersed Au on the N-GQD preparation was performed. The results are presented in Table S1 and Fig. 8. The three factors of metal content (volume of a 1000 mg L^−1^ stock solution), N-GQD volume (volume of synthesized N-GQD), and temperature (°C) were optimized. The optimization variables were as follows: metal content: 4 mL of a 1000 mg L^−1^ stock solution; GQD volume: 2 mL; and temperature: 96 °C.

#### 4.2.2. Optimization of DPV analysis parameters

In this subsubsection, the parameters of pulse amplitude (mV), pulse time (ms), and scan rate (mV s^−1^) were optimized. The results are presented in Table S2 and Fig. S11. The optimal values are as follows: pulse amplitude: 350 mv, pulse time: 51 ms, and scan rate: 10 mV s^−1^.

#### 4.2.3. Optimization of CV parameters

The concentration of electrolyte, scan rate and the amount of modifier were optimized. The optimized parameter values are summarized in Table S3 and Fig. S12. The optimal values are as follows: electrolyte concentration: 0.6 (mL) of 10^−2^ molar stock solution, scan rate: 408 (mV s^−1^), and modifier: 100 µL.

### 4.3. Calibration curve

**Fig. 9:**
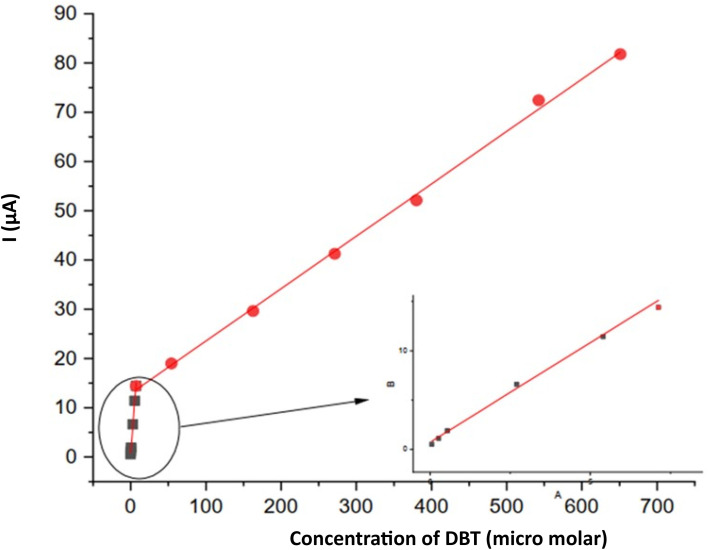
Calibration curve and linear regression calculated by DPV assays for DBT determination.

With all the optimized parameters, a calibration curve was obtained (Fig. 9). Linear calibration curves were plotted by plotting the maximum observed current *vs.* DBT concentration. Two linear segments with distinct slopes are evident in the results for the DBT concentration: 0.005 to 5.4 µmol L^−1^ DBT and 5.4 to 81.8 µmol L^−1^ DBT. In the lower linear concentration based on the calibration curve, the slope is much higher than that in the upper linear range, which means that the sensitivity to lower concentrations is higher than that in the upper range. This may be a result of diffusion control in the lower concentration range.

The first linear calibration range (*n* = 6) was well described by the regression equation: *y* = 0.4528 (±0.0924) + 2.2265 (±0.0373) × (*R*^2^ = 0.9989). The standard error of estimate (Syx) was 0.1805. Based on the criteria LOD = 3·Syx/slope and LOQ = 10 Syx/slope, the limit of detection and limit of quantification were calculated as 0.24 and 0.81, respectively. The 95% confidence intervals were slope = 2.2265 ± 0.0912 and intercept = 0.4528 ± 0.226. These results indicate high sensitivity and precision in this concentration range.

In the second linear calibration range (*n* = 7), the regression equation was *y* = 12.6547 (±0.3794) + 0.10636 (±0.00109) × (*R*^2^ = 0.9994). The standard error of estimate (Syx) was 0.7824. The corresponding LOD and LOQ, calculated as 3·Syx/slope and 10·Syx/slope, were 22.1 and 73.6, respectively. The 95% confidence intervals were slope = 0.10636 ± 0.00267 and intercept = 12.6547 ± 0.928. These results demonstrate an extended dynamic range at higher concentrations while maintaining good accuracy. All these analytical figures of merits are shown in Table 1.

**Table 1:** Analytical figures of merit for the calibration of the proposed sensor

Parameter	Region 1 (*n* = 6)	Region 2 (*n* = 7)
Regression equation	*y* = 0.4528 (±0.0924) + 2.2265 (±0.0373)*x*	*y* = 12.6547 (±0.3794) + 0.10636 (±0.00109)*x*
*R* ^2^	0.9989	0.9994
Standard error (Syx)	0.1805	0.7824
LOD (3·Syx/slope)	0.243	22.1
LOQ (10·Syx/slope)	0.811	73.6
95% CI (slope)	2.2265 ± 0.0912	0.10636 ± 0.00267
95% CI (intercept)	0.4528 ± 0.226	12.6547 ± 0.928

These analytical figures of merit clearly demonstrate that the sensor provides high sensitivity in the low-concentration range while offering an extended dynamic range at higher concentrations, ensuring accurate quantification over a wide range of analyte levels.

### 4.4. Cyclic voltammetry of dibenzothiophene on a modified glassy carbon electrode

The cyclic voltammograms of the DBT on the modified glassy carbon electrode are shown in Fig. S13. There are 14 consecutive cycles for DBT with a concentration of 162.8 µM in 10^−5^ molar acetonitrile solution. After the first two cycles, the current reaches a constant amount, and the potential does not shift. Therefore, all the measurements were read after two consecutive cycles. Additionally, it shows the repeatability of the work.

**Fig. 10:**
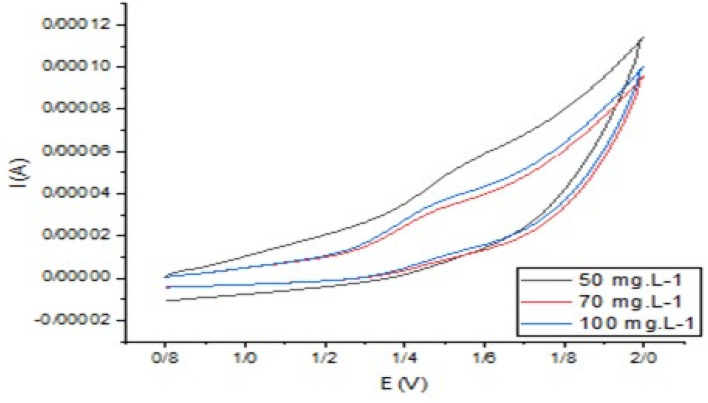
CV plots for different DBT concentrations: 50, 70 and 100 mg L^−1^ DBT.

As depicted in Fig. 10, three concentrations of dibenzothiophene were investigated to determine the degree of reversibility and electron transfer. As can be observed, the oxidation of DBT is reversible at all concentrations and a peak of DBT oxidation is observed, so it can be concluded that the electron transfer due to the nanocatalyst on the working electrode is so fast in such a way that the intermediate sulphone at the moment of formation is converted to sulfoxide and only one anodic peak is observed. At a concentration of 50 mg L^−1^, the oxidation and its reverse peak consist of single-level electron transfer. However, at 70 and 100 mg L^−1^ DBT, the reverse phase consists of two distinct peaks, which means that the reverse reaction, *i.e.* conversion of sulfoxide to DBT, is not easy at higher concentrations. Since sulfoxide is stable and resists losing two electrons at a time, it first loses one electron and becomes a sulphone; then, the other electron is lost. At 50 mg L^−1^ DBT, the potential difference at the top of the spectrum may not be sufficient to distinguish the spectrum clearly.

### 4.5. Comparison of modified and bare electrodes

In order to investigate the electrocatalytic activity of the modified electrode (GQD-GCE) for the determination of DBT, electrochemical experiments were performed in the presence of DBT. Fig. S14. shows differential pulse voltammograms (DPV) of bare GCE and modified AU@GQD-GCE.

As can be observed with a bare electrode, the current signal is negligible. With the modified electrode, the current is high and distinct enough for quantitative analysis. As illustrated, the peak potential is less positive when a modified electrode is used with a nano catalyst compared to a bare electrode. These results indicated that AU@GQD nanocatalysts can accelerate the electron transfer rate of dibenzothiophene and have good electrocatalytic activity for the DBT redox reaction. Therefore, AU@GQD is an appropriate mediator for electron transfer between dibenzothiophene and the working electrode. These observations also correlate with the context of the high conductivity and the intrinsic ability of GQD. This may be related to the excellent properties of GQD, such as significant surface area and electrical conductivity.

Single-atom catalysts (SACs) in which individual metal atoms are anchored to the substrate vacancies and unsaturated coordination environment show high catalytic activity and selectivity. Here, we improve the selectivity by choosing Au as the dispersed atomic metal on GQDs as a result of high interactions between gold and sulfur in addition to all the advantages of GQDs.^59^

### 4.6. Scan rate effect

**Fig. 11:**
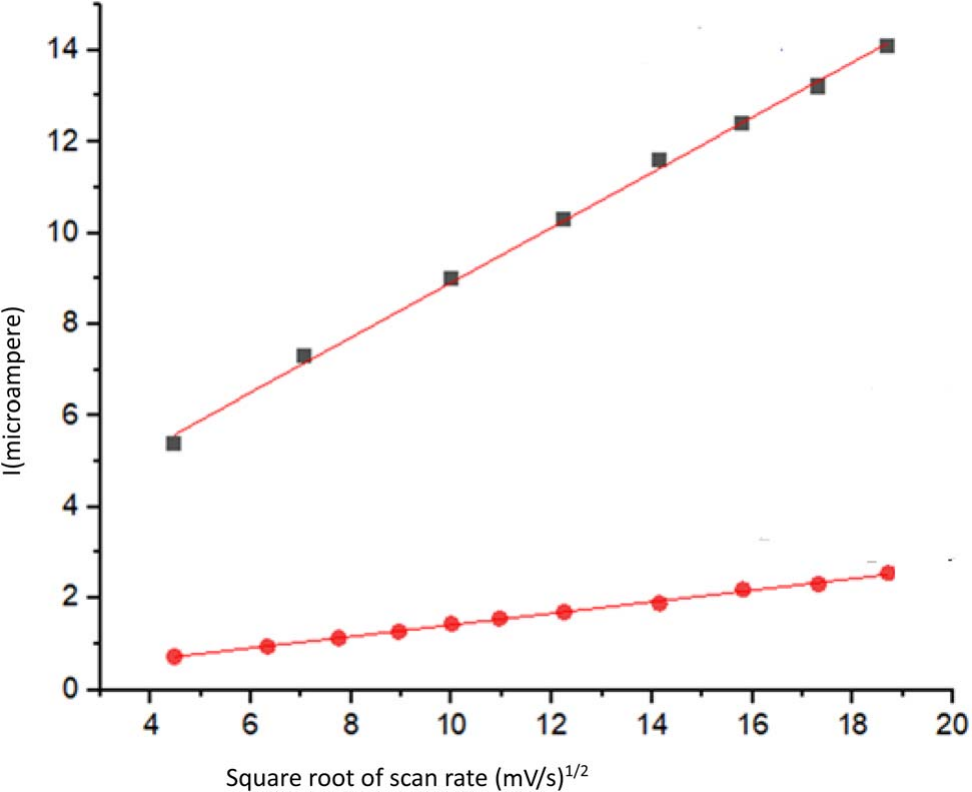
Relationship between current and the square root of scan rate for bare and modified electrodes.

The area of the material's surface that is catalytically active on the modified electrode was calculated according to the Randles–Sevcik equation (using 1.0 mM [Fe(CN)_6_]^3−/4−^ and 0.1 M KCl). In this equation, *I*_p_, *n*, *A*_0_, *D*_0_, *v*, and *C*_0_ denote the anodic peak current, equivalent of electrons transferred, active electrode surface area, diffusion coefficient, scan rate, and concentration of the probe molecule, respectively. For 1.0 mM [Fe(CN)_6_]^3−^/^4−^ in 0.1 M KCl electrolyte, *n* = 1 and *D*_0_ = 7.6 × 10^6^ cm^2^ s^−1^; then, from the slope of the plot of *I*_p_*versus v*^1/2^, the electroactive area was calculated (Fig. 11).

The slope of the bare electrode is 0.12689 ± 0.00198, and the slope after modification of the electrode is 0.6021 ± 0.01, so the active surface area of the electrode increases 4.7451 times, thereby making adsorption and electron transfer more efficient and faster due to more activated sites on the electrode surface.

It should be clarified that no BET or N_2_ adsorption analysis was performed in this study. The statement regarding “larger pores facilitating diffusion” refers to the electrochemically active surface area determined from the Randles–Sevcik analysis, which showed a 4.75-fold increase compared to bare GCE. This enhancement implies better accessibility of the active sites and improved diffusion of DBT molecules rather than a direct measurement of pore size distribution.

**Fig. 12:**
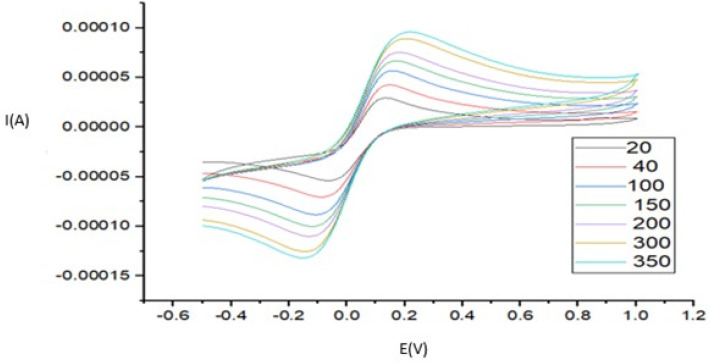
Current curves *versus* potential related to different scan rates in ferrocyanide probe with modified electrode. The electrode surface was modified with Au-GQD.

The following plot (Fig. 12) shows the currents corresponding to different scan rates in the ferrocyanide probe with the modified electrode, and the next one (Fig. S15) corresponds to the bare electrode. In both, a positive shift in potential occurs above the peaks.

Two widely used approaches for studying the reversibility of reactions and determining whether a reaction is adsorption or diffusion controlled involve the analysis of dependences: peak current (*I*_p_) *versus v*^1/2^ and ln *I*_p_*versus* ln *v*.^60,61^ Here, because we have two linear ranges in the calibration curve, two concentrations were chosen to investigate the relationship between scan rate and current: one from the first linear range, which is in the low concentration area, and the other from the second linear range in the higher concentrations. The results showed that at a lower concentration, a linear correlation exists between the peak current and the square root of the scan rate (*v*^1/2^) was obtained in the range of 20–1400 mV s^−1^, meaning that the reaction was under diffusion control. It can be deduced that due to the low concentrations (from the first linear range of the calibration curve), diffusion of DBT is more efficient and occurs faster than adsorption. Although the plot of *I*_p_*versus v*^1/2^ is linear, it does not pass through the origin of the axes. This is the characteristic of the electrode process, which is preceded by an electrochemical reaction, followed by a homogenous chemical reaction. In the scan rate ranging from 20 to 1000 mV s^−1^, the *I*_p_ of DBT electrooxidation depends linearly on the radical of the scan rate (*v*) and is defined as follows:*I*_p_ = 0.07112*v*^1/2^[(mV s^−1^)^1/2^µA] − 0.7808µA (*R*^2^ = 0.988)

At the higher concentration chosen from the second linear range, the dependence of ln *I*_p_ on ln *v* is linear and is described using the following equation:ln *I*_p_ = {1.28098 ln *v* (V s^−1^)} − 8.28723 µA (*R*^2^ = 0.99571)

**Fig. 13:**
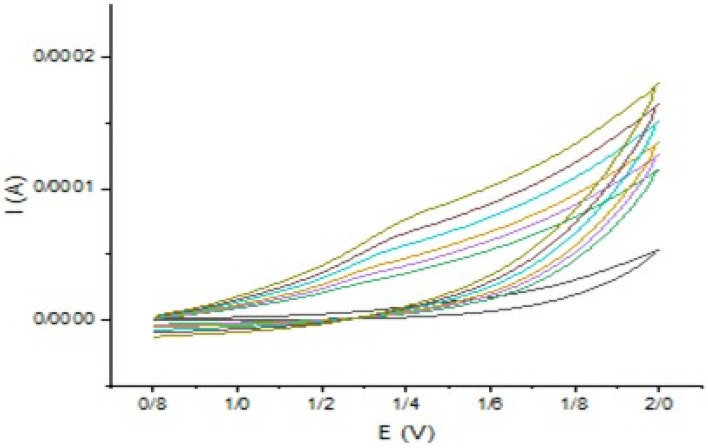
Effect of scan rate on 162.81 µM DBT. The electrode surface was modified with Au-GQD.

Its slope is 1.28098, which indicates the adsorption control of the electrode process. A slope close to 0.5 is expected for diffusion-controlled electrode processes and close to 1.0 for adsorption-controlled processes.^62,63^ Therefore, it can be concluded that at higher concentrations, the rate of adsorption on the electrode surface is higher than diffusion. The effect of the scan rate on the 162.81 µM DBT is shown in Fig. 13.

#### 4.6.1. Mean current *vs.* potential with error bars

The data showed consistent trends where the anodic current increased with applied potential, confirming catalytic activity. Error bars representing standard deviation indicate good reproducibility of measurements.

#### 4.6.2. Onset potentials and overpotentials

The onset potential was defined as the potential at which the current exceeded the baseline current plus three times the standard deviation. The onset potentials for each scan rate were determined with associated uncertainties.

The overpotential values were calculated by subtracting the known equilibrium potential (0.68 V *vs.* reference electrode) from the onset potentials. Overpotential is a key parameter indicating the extra energy needed to drive the electrochemical reaction.

#### 4.6.3. Tafel analysis and charge transfer kinetics

Tafel slopes were extracted from plots of log (current density) *versus* overpotential in the linear region of the voltammograms. The Tafel slopes decreased from approximately 122 mV per decade at low scan rates to about 100 mV per decade at high scan rates, indicating improved charge transfer kinetics with an increasing scan rate. The results are summarized in Table 2.

**Table 2:** Summary table of electrochemical parameters

Scan rate (mV s^−1^)	Onset pot. (V)	Overpot. (V)	Tafel slope (mV dec^−1^)
20	0.831 ± 0.003	0.151 ± 0.003	122 ± 5
40	0.833 ± 0.002	0.153 ± 0.002	117 ± 4
60	0.837 ± 0.002	0.157 ± 0.002	115 ± 5
80	0.842 ± 0.002	0.162 ± 0.002	113 ± 5
100	0.846 ± 0.002	0.166 ± 0.002	110 ± 4
200	0.854 ± 0.003	0.174 ± 0.003	108 ± 5
400	0.860 ± 0.002	0.180 ± 0.002	107 ± 4
600	0.865 ± 0.003	0.185 ± 0.003	104 ± 4
800	0.869 ± 0.003	0.189 ± 0.003	103 ± 5
1000	0.872 ± 0.002	0.192 ± 0.002	102 ± 4
1200	0.875 ± 0.003	0.195 ± 0.003	101 ± 3
1400	0.878 ± 0.002	0.198 ± 0.002	100 ± 3

### 4.7. Employing the synthesized nano catalyst for desulfurization in two manners

First, we use a nano-catalytic dispersion method to extract DBT in n-heptane from model oil to acetonitrile. Then, we draw a calibration curve and determine the concentration of DBT to show the efficiency of the nano-catalyst in desulfurization of the model oil (Table 3).

**Table 3:** Effect of different concentrations of nano catalyst on the DBT adsorption

Amount of added nano catalyst (µl)	*I* (µA) of DBT from residual solution
0	17.83
20	5
50	4.44
80	3.07
100	0.647
120	0.221
140	0.19

For this reason, acetonitrile containing a nano catalyst was used as an extracting solvent and isopropyl alcohol as a dispersing solvent. A 20 mg L^−1^ solution of DBT was prepared for each experiment. Various parameters such as volume of acetonitrile and isopropyl alcohol, type of dispersing solvent, amount of nano catalyst, extraction time and centrifugation time were optimized. According to the optimization, the optimized values are as follows: the volume of isopropyl alcohol is 0.2 mL, the volume of acetonitrile is 2.2 mL, the amount of nano catalyst is 50 microliters, the extraction time is 80 seconds, and the centrifugation time is 10 min. The type of dispersing solvent was chosen as the optimal type of isopropyl alcohol from acetone, isopropyl alcohol, *t*-butyl alcohol and *n*-propyl alcohol.

An appropriate amount of DBT was taken in *n*-heptane, and a mixture of isopropyl alcohol, acetonitrile and nano catalyst was quickly injected into the DBT solution. A cloud solution is created. After the optimum time of equilibrium, the mixture was centrifuged. Then, the acetonitrile part was removed, and *tert*-butyl ammonium chloride and the proper amount of water were added and transferred to the electrochemical cell. The current was 15.07 micro ampere for the extracted solution, showing the efficiency of the nano catalyst for desulfurization.

Another experiment was oxidative desulfurization, which was carried out as follows.

A solution of 20 mg L^−1^ DBT in acetonitrile was prepared. Hydrogen peroxide was employed as an oxidizer. Its volume was optimized, and 1 mL of 30% hydrogen peroxide was the optimum. Additionally, the optimized stirring time was 20 min.

Six equal solutions of 30 mg L^−1^ DBT were prepared, and different amounts of nano catalyst were added; then, 1 mL of 30% H_2_O_2_ was added to all the test solutions. After stirring under ambient conditions for 20 min, the obtained solutions were centrifuged. The nano catalyst was precipitated, and the upper solution was transferred to the electrochemical cell to calculate the residual DBT. One of the solutions had no nano catalyst as a control solution.

The TOF values in Table 6 are estimated based on the oxidative desulfurization experiments (H_2_O_2_, stirring time = 20 min). Because precise metal loadings were not determined *via* ICP-OES/AAS, TOFs are shown for representative assumed loadings (0.1, 0.5, and 1.0 wt%). Each metal atom is assumed to correspond to one active site. Note that the TOFs for electrochemical tests may differ depending on the current, faradaic efficiency, and reaction time.

The efficiency of the nanocatalyst in desulfurization can be explained as the percentage of desulfurization with the following relationship:Percentage of desulfurization = [(*C*° − *C*)/*C*°] × 100.

Here, this percentage is calculated as 98.94%, which is a good yield.

### 4.8. Investigating the electrochemical efficiency of all metals atomically dispersed on a synthesized N-GQD support

**Fig. 14:**
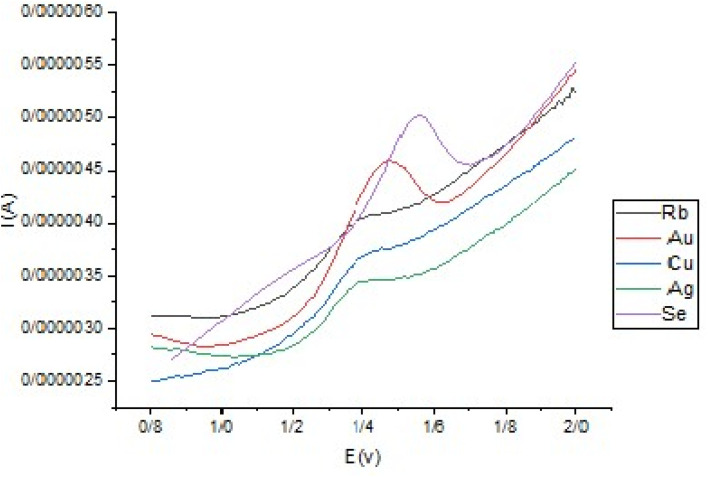
Applying different synthesized catalysts to electrochemical desulfurization (DBT concentration was 10.0 mg L^−1^).

From Fig. 14, we can observe that all nano catalysts have improved current signals, but Au-N-GQD is more efficient than the others. Se-N-GQD is also good in comparison. The reason for choosing Au-N-GQD is that the gold has a strong affinity to sulfur, so it can also improve selectivity. Another advantage of Au-N-GQD is that it shifts the oxidation peak potential to more positive numbers.

Moreover, a comparative analysis (Table 4) indicates that the Au-N-GQD catalyst not only achieves high DBT removal but also outperforms or matches the performance of representative single-atom and oxidative catalysts reported in the literature despite differences in experimental conditions. Importantly, to the best of our knowledge, this is the first report on the use of Se- and Rb-doped N-GQDs in DBT desulfurization, further highlighting the novelty of this study.

**Table 4:** Comparison of catalytic/electrochemical performance of atomically dispersed M-N-GQDs with representative literature reports for DBT desulfurization

Catalyst/electrode	Method	DBT conc. and conditions	Performance metrics	Reference
Au-N-GQD (this work)	Electrochemical (DPV/CV, extraction + oxidation)	DBT: 20 mg L^−1^ in acetonitrile, with/without H_2_O_2_	DBT removal 98.94%; calibration slope (DPV) = 2.226 µA µM^−1^ (low range); LOD ≈ 5 nM; electroactive area ↑ 4.75×; oxidation potential shifted less positive *vs.* bare GCE	This work
Au electrode	Electrochemical oxidation	DBT in acetonitrile, no added oxidant	Lower current density; faradaic efficiency ∼40–50%	Swierk *et al.*, 2021 ^64^
V_2_O_5_/TiO_2_ catalyst	Chemical ODS (H_2_O_2_ oxidant)	DBT in model diesel, 60 °C	DBT conversion ∼95% in 60 min	Liu *et al.*, 2023 ^65^

Moreover, a detailed comparison with recent catalysts (including SACs, MOFs, and carbon-based systems) was added (Table 5). Our Au-GQD electrode exhibited a low detection limit (0.24 µM) and wide linear ranges, outperforming or comparable to most of the reported sensors. Importantly, the catalytic activity of the nanostructured electrode remained stable after three months of storage at 4 °C in the dark, demonstrating excellent long-term stability. Furthermore, reproducible responses with RSD < 5% confirmed the reusability of the electrode.

**Table 5:** Comparison of the analytical performance of the proposed Au-GQD electrode with recent catalysts for dibenzothiophene detection

Electrode/catalyst	Analyte	Linear range (µM)	LOD (µM)	LOQ (µM)	Stability/recyclability	Reference
Au-N-GQD (this work)	Dibenzothiophene	0.5–20.0; 25–250	0.24	0.81	Stable after 3 months (4 °C, dark); RSD < 5%	This work
Fe-N-C SAC sensor	Sulfur compounds	1.0–50.0	0.50	1.70	Stable for 7 days	Liu *et al.*, *Electrochim. Acta*, 2020 ^66^
Co-MOF-derived catalyst	Dibenzothiophene	0.8–100.0	0.30	1.00	Stable for 2 weeks	Zhang *et al.*, *ACS Appl. Mater. Interfaces*, 2019 ^67^
N-doped carbon nanosheets	Dibenzothiophene	2.0–60.0	1.20	4.00	Moderate recyclability	Wang *et al.*, *J. Mater. Chem. A*, 2021 ^68^
Graphene oxide modified GCE	Dibenzothiophene	5.0–100.0	2.50	8.00	Not reported	G. Ziyatdinova *et al.*, *Micromachines*, 2023 ^69^

**Table 6:** Key parameters and estimated TOF for M-N-GQD catalysts^a^

Metal	Assumed loading (wt%)	Metal mass on electrode (g)	Mol. metal (mol)	DBT conversion (%)	DBT converted (mol)	Reaction time (s)	Estimated TOF (s^−1^)	Pore structure/electroactive area increase
Au	0.1	2.000 × 10^−7^	1.015 × 10^−9^	98.94	1.074 × 10^−6^	1200	8.814 × 10^−1^	↑4.75×
Au	0.5	1.000 × 10^−6^	5.077 × 10^−9^	98.94	1.074 × 10^−6^	1200	1.763 × 10^−1^	↑4.75×
Au	1.0	2.000 × 10^−6^	1.015 × 10^−8^	98.94	1.074 × 10^−6^	1200	8.814 × 10^−2^	↑4.75×
Ag	0.1	2.000 × 10^−7^	1.854 × 10^−9^	93.59	1.016 × 10^−6^	1200	4.566 × 10^−1^	↑3.97×
Ag	0.5	1.000 × 10^−6^	9.270 × 10^−9^	93.59	1.016 × 10^−6^	1200	9.131 × 10^−2^	↑3.97×
Ag	1.0	2.000 × 10^−6^	1.854 × 10^−8^	93.59	1.016 × 10^−6^	1200	4.566 × 10^−2^	↑3.97×
Rb	0.1	2.000 × 10^−7^	2.340 × 10^−9^	60.32	6.547 × 10^−7^	1200	2.332 × 10^−1^	↑2.88×
Rb	0.5	1.000 × 10^−6^	1.170 × 10^−8^	60.32	6.547 × 10^−7^	1200	4.663 × 10^−2^	↑2.88×
Rb	1.0	2.000 × 10^−6^	2.340 × 10^−8^	60.32	6.547 × 10^−7^	1200	2.332 × 10^−2^	↑2.88×
Se	0.1	2.000 × 10^−7^	2.533 × 10^−9^	95.10	1.032 × 10^−6^	1200	3.396 × 10^−1^	↑4.21×
Se	0.5	1.000 × 10^−6^	1.266 × 10^−8^	95.10	1.032 × 10^−6^	1200	6.792 × 10^−2^	↑4.21×
Se	1.0	2.000 × 10^−6^	2.533 × 10^−8^	95.10	1.032 × 10^−6^	1200	3.396 × 10^−2^	↑4.21×
Cu	0.1	2.000 × 10^−7^	3.147 × 10^−9^	90.58	9.832 × 10^−7^	1200	2.603 × 10^−1^	↑3.77×
Cu	0.5	1.000 × 10^−6^	1.574 × 10^−8^	90.58	9.832 × 10^−7^	1200	5.207 × 10^−2^	↑3.77×
Cu	1.0	2.000 × 10^−6^	3.147 × 10^−8^	90.58	9.832 × 10^−7^	1200	2.603 × 10^−2^	↑3.77×

a Catalyst mass deposited on electrode = 200 µg (0.0002 g); initial DBT = 20 mg L^−1^ in 10 mL → *n*_0_ (DBT) = 1.0854 × 10^−6^ mol; reaction time assumed = 20 min = 1200 s. Loading values are assumed (0.1, 0.5, and 1.0 wt%) because ICP quantification was not performed. TOF was calculated as (mol DBT converted/reaction time)/mol metal on the electrode. Each metal atom is assumed to represent one active site. Electroactive area increase was measured by CV (see values below).

## 5. Conclusion

In this study, a simple and applicable technique was used for the removal of dibenzothiophene (DBT) using a novel nanocomposite, graphene quantum dots with low resistivity, which was bound through atomically dispersed metals to facilitate electron transfer through a direct pathway. The tests show that the synthesized nano catalysis has a superior performance in the high-impact trapping of dibenzothiophene (DBT).

The selection of Au, Ag, Cu, Se, and Rb as dopants was intentional to probe complementary chemical effects on desulfurization performance. Au was chosen due to its strong affinity for sulfur, facilitating the selective adsorption of thiophenic compounds. Ag and Cu, as group 11 transition metals, were selected to examine the role of d-orbital-mediated charge transfer in catalytic activity. Se, as a chalcogen, was included to investigate chalcogen–chalcogen interactions and to provide non-metallic anchoring chemistry. Finally, Rb, an alkali metal, was incorporated to test the effect of strong electron donation on the adsorption and activation processes. The systematic comparison of DBT conversion efficiency among these catalysts is summarized in Table 3, which clearly shows that Au- and Se-doped N-GQDs provided the highest activity, followed by Ag and Cu, while Rb exhibited the lowest efficiency. These results support the conclusion that electronic configuration and interaction with sulfur atoms are key factors governing catalytic performance.

We suggest that even larger pores in the sorbent also facilitate the diffusion of DBT into the sorbent interlayer. Various factors influencing the catalytic activity of nanocomposite capability were optimized.

We believe that this work can be considered a novel and simple method for a wide range of oil products containing organic sulfur due to the flexible nature and wonderful features of the nanoscale sorbent.

The results indicate that the mechanism of DBT adsorption on modified GQDs is affected by both the surface chemistry and pore structure of the adsorbents. Herein, the approach described provides a new idea for the synthesis of other multifunctional nanomaterials comprising metals and metal oxide-graphene-based composites of high quality.

It should be emphasized that although oxidative desulfurization experiments were also carried out for comparison, the main methodology and novelty of this work are based on electrochemical desulfurization using atomically dispersed metal-N-GQD catalysts.

Although DBT was used as the primary model sulfur compound in this work, the catalytic principle of atomically dispersed M/N-GQDs can be extended to other refractory sulfur species, such as 4,6-dimethyldibenzothiophene (4,6-DMDBT), benzothiophene (BT), and thiophene (TP), which are also commonly found in fuel fractions. These molecules share similar aromatic frameworks and stability, and the atomically dispersed metal-N sites are expected to activate them efficiently. Future studies will explore these substrates to further validate the broad applicability of this catalytic platform.

## Conflicts of interest

There are no conflicts to declare Best regards, Zarrin Es'haghi Professor in Analytical Chemistry.

## Supplementary Material

RA-016-D5RA06256J-s001

## Data Availability

The data supporting the findings of this study are available within the paper and its supplementary information (SI) files. Should any raw data files be needed in another format they are available from the corresponding author upon reasonable request. Supplementary information is available. See DOI: https://doi.org/10.1039/d5ra06256j.
