# Supplementary material for: Atomically dispersed high-loading metals and metalloids on a graphene quantum dot-support for selective electrochemical desulfurization
Source: RSC Adv. 2026 Jan 6;16(2):1956–76. doi: 10.1039/d5ra06256j (PMC12772627; doi:10.1039/d5ra06256j)
Supplement: RA-016-D5RA06256J-s001 [file RA-016-D5RA06256J-s001.pdf]

This journal is © The Royal Society of Chemistry 2025

Supplementary file

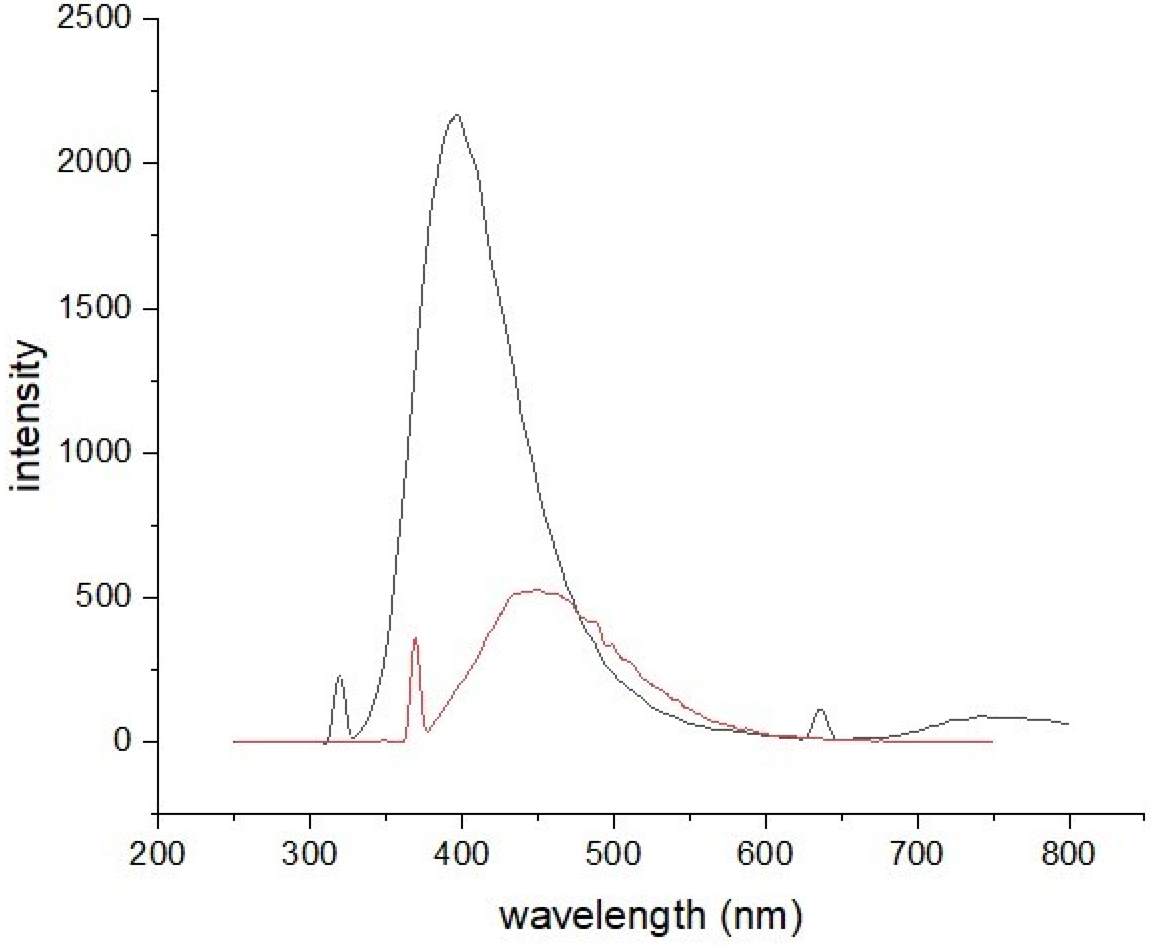

Figure S1.

———— Fluorescence spectra of GQD synthesized via glucose precursor

———— Fluorescence spectra of GQD synthesized via citric acid precursor.

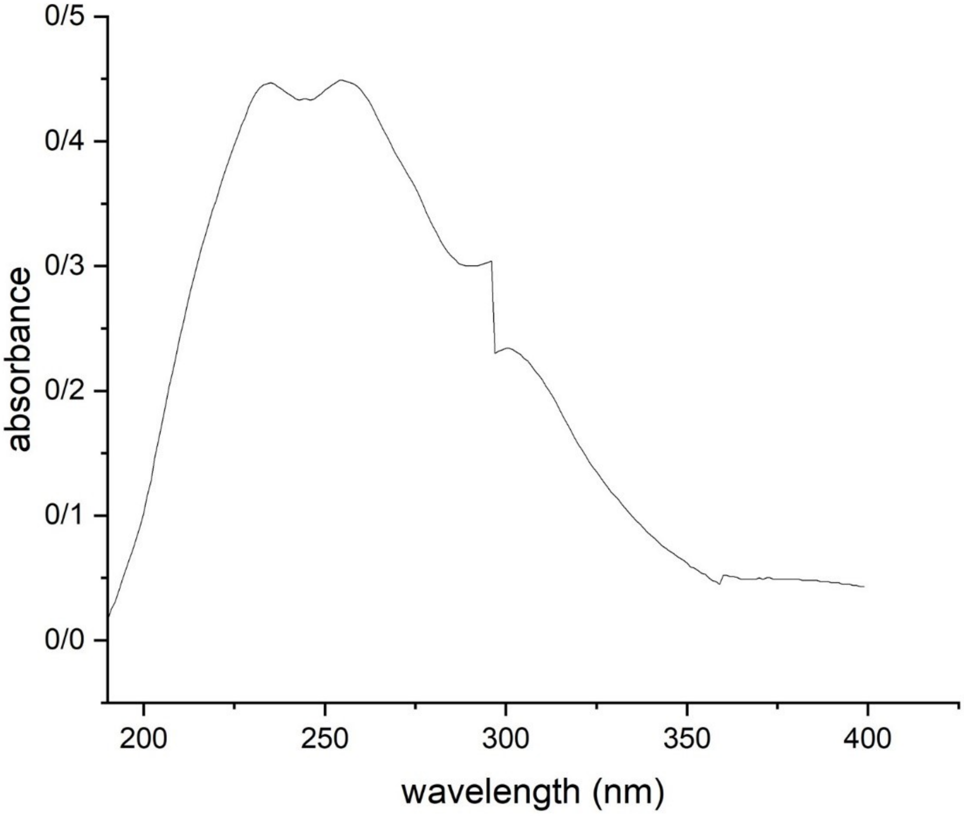

Figure S2. UV-Vis spectra of GQD synthesized based on glucose.

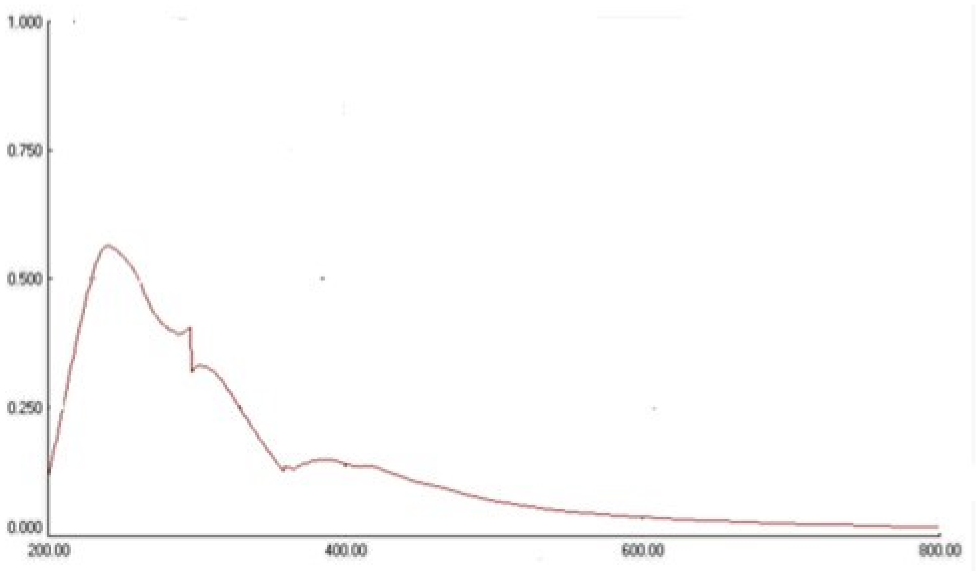

Figure S3. UV-Vis spectra of GQD synthesized based on citric acid.

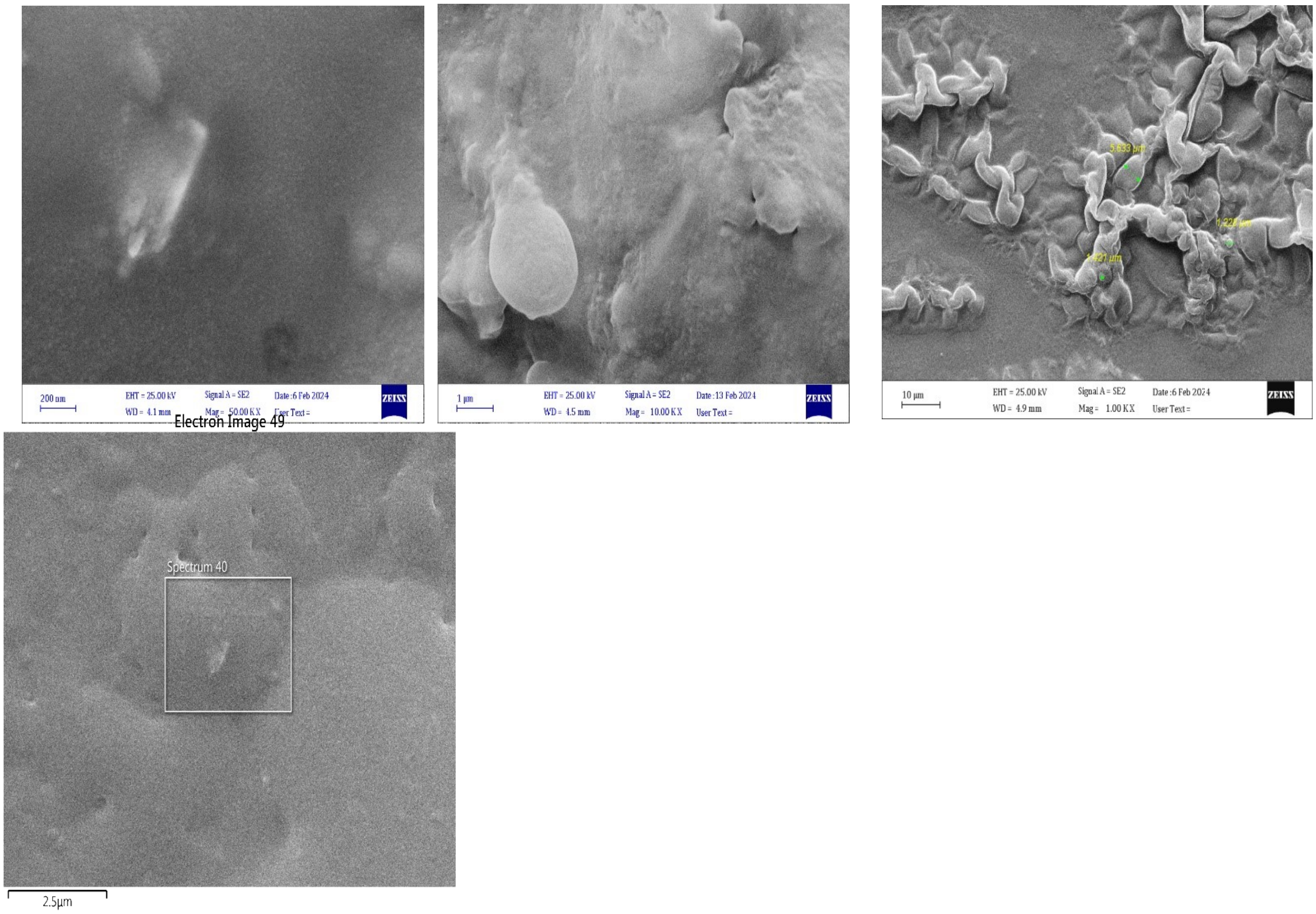

Figure S4. SEM images of synthesized GQDs based on glucose with different magnifications.

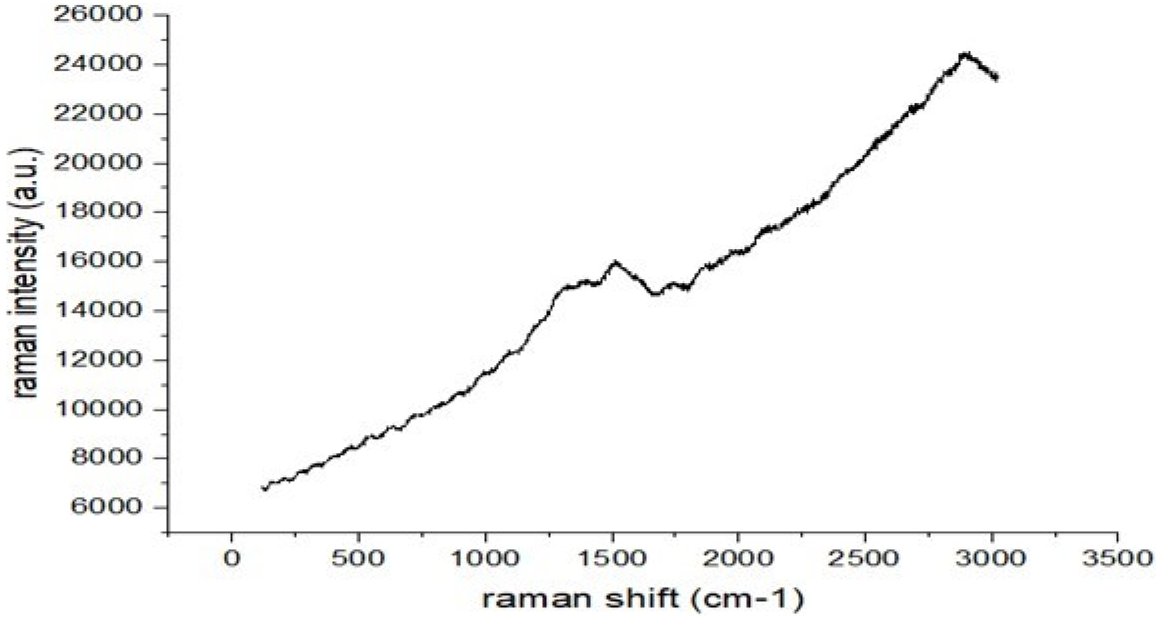

Figure S5 Raman spectra of GQD synthesized from glucose

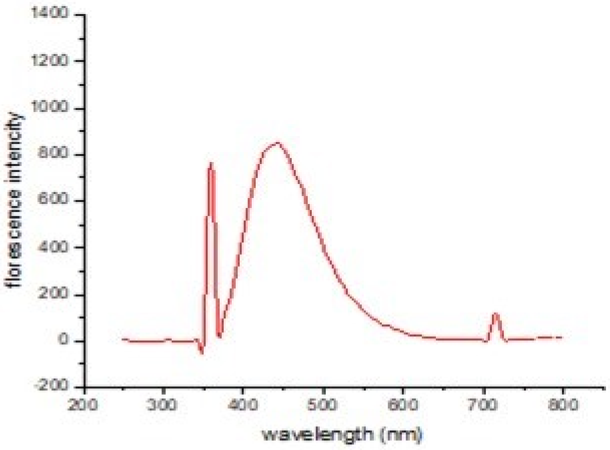

(a) Ag

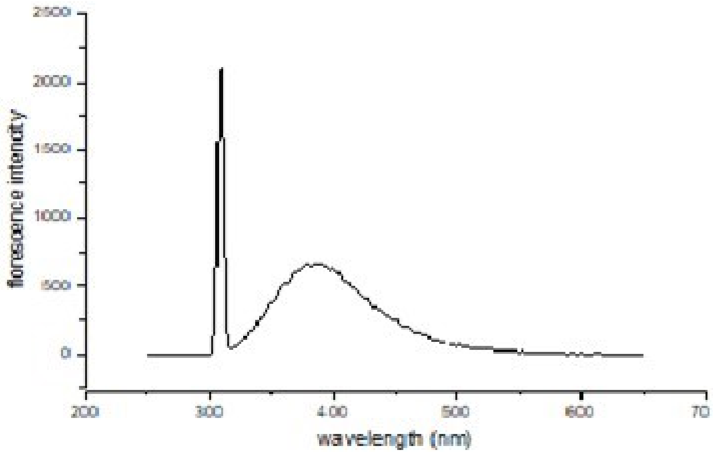

(b)Rb

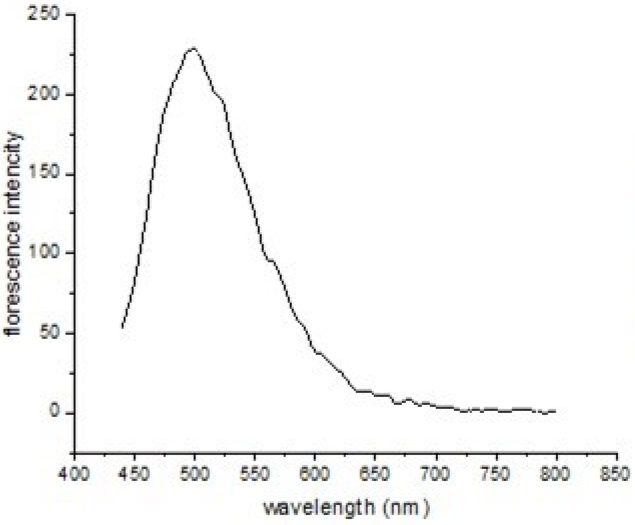
florescence intencity
wavelength (nm)

(c)Cu

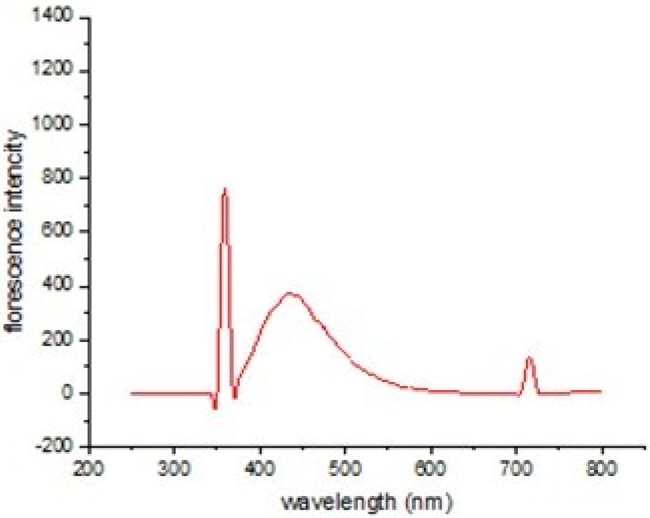
florescence intencity
wavelength (nm)

(d)Se

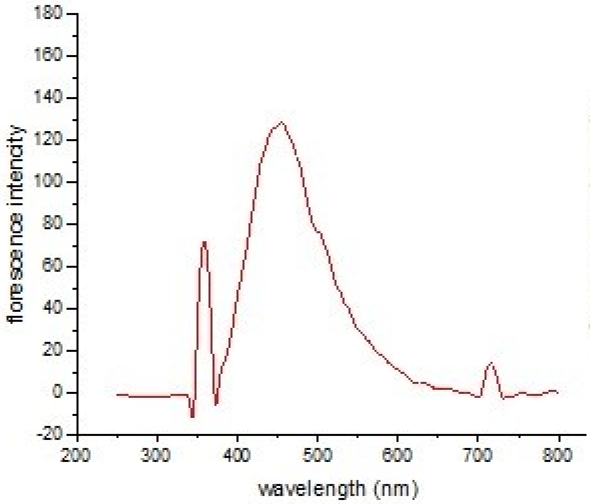
florescence intencity
wavelength (nm)

(e)Au

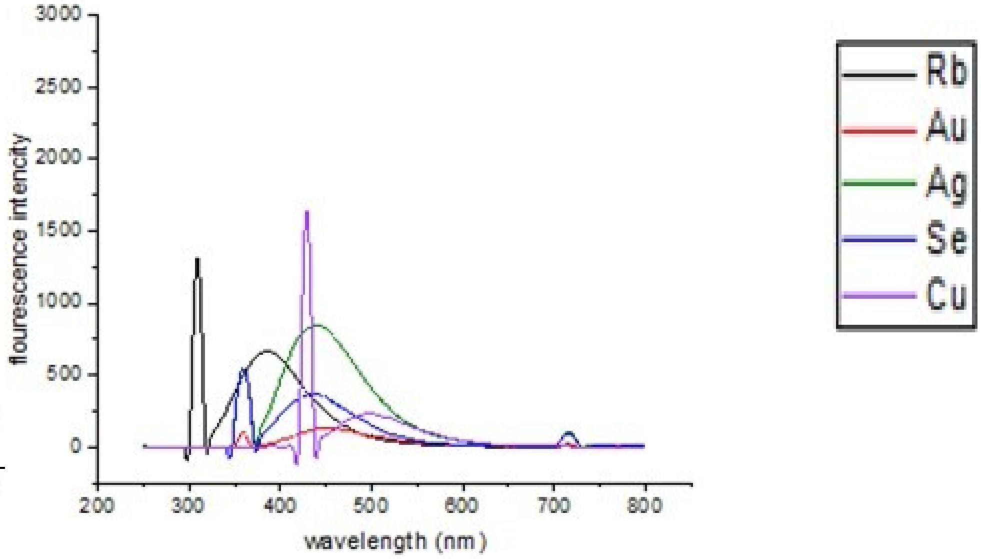
flourescence intencity
wavelength (nm)
Rb
Au
Ag
Se
Cu

(f)

Figure S6. a-e) The fluorescence spectrum of metals atomically anchored on glucose based GQD, **f)** all spectra at one plot for better comparing.

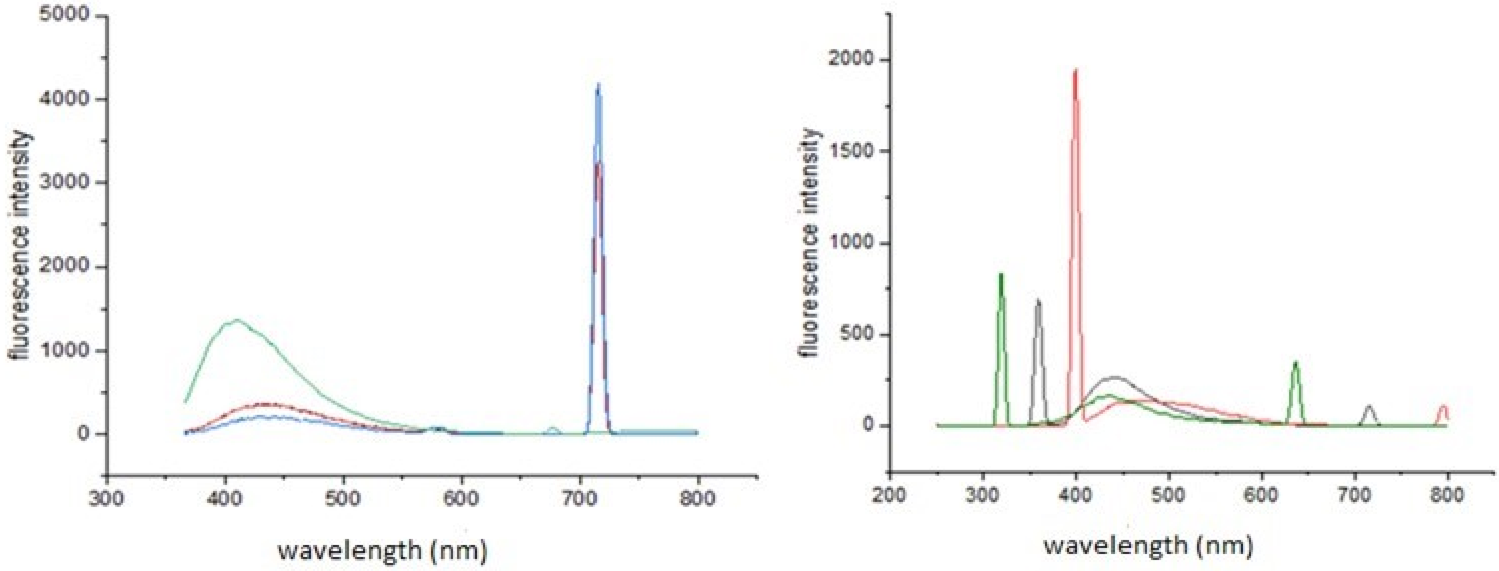

Figure S.7. a) The fluorescence spectra of glucose based GQD and Cu-dopped GQDs by using different amounts of copper: 0, 10, 20 and 40 mg. L^{-1} respectively from up to down. b) The fluorescence spectra of citric acid-based GQDs (black) and Cu-dopped GQDs with 40 mg. L^{-1} (red) and 50 mg. L^{-1} (green).

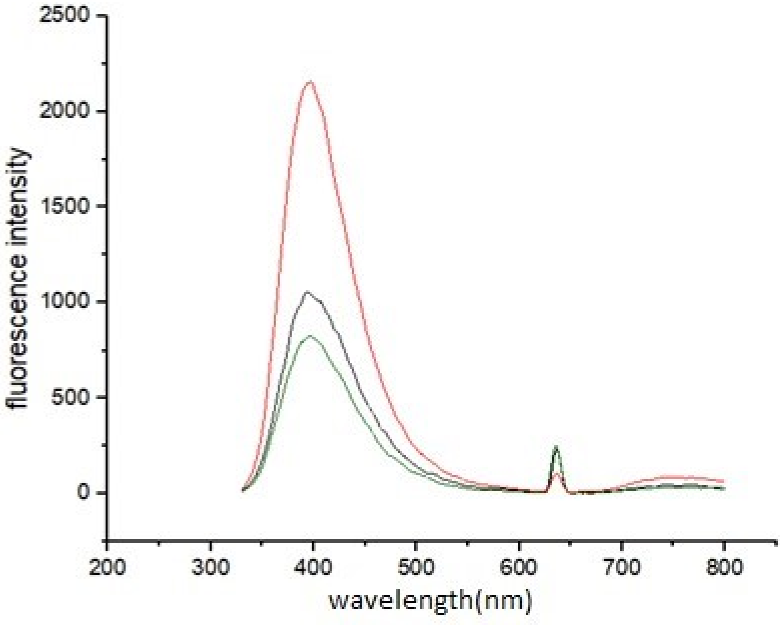

Figure S.8. The fluorescence spectra of GQD from glucose and Rb-GQD, Rb concentrations are: 0, 10 and 40 mg. L^{-1} respectively from up to down spectra.

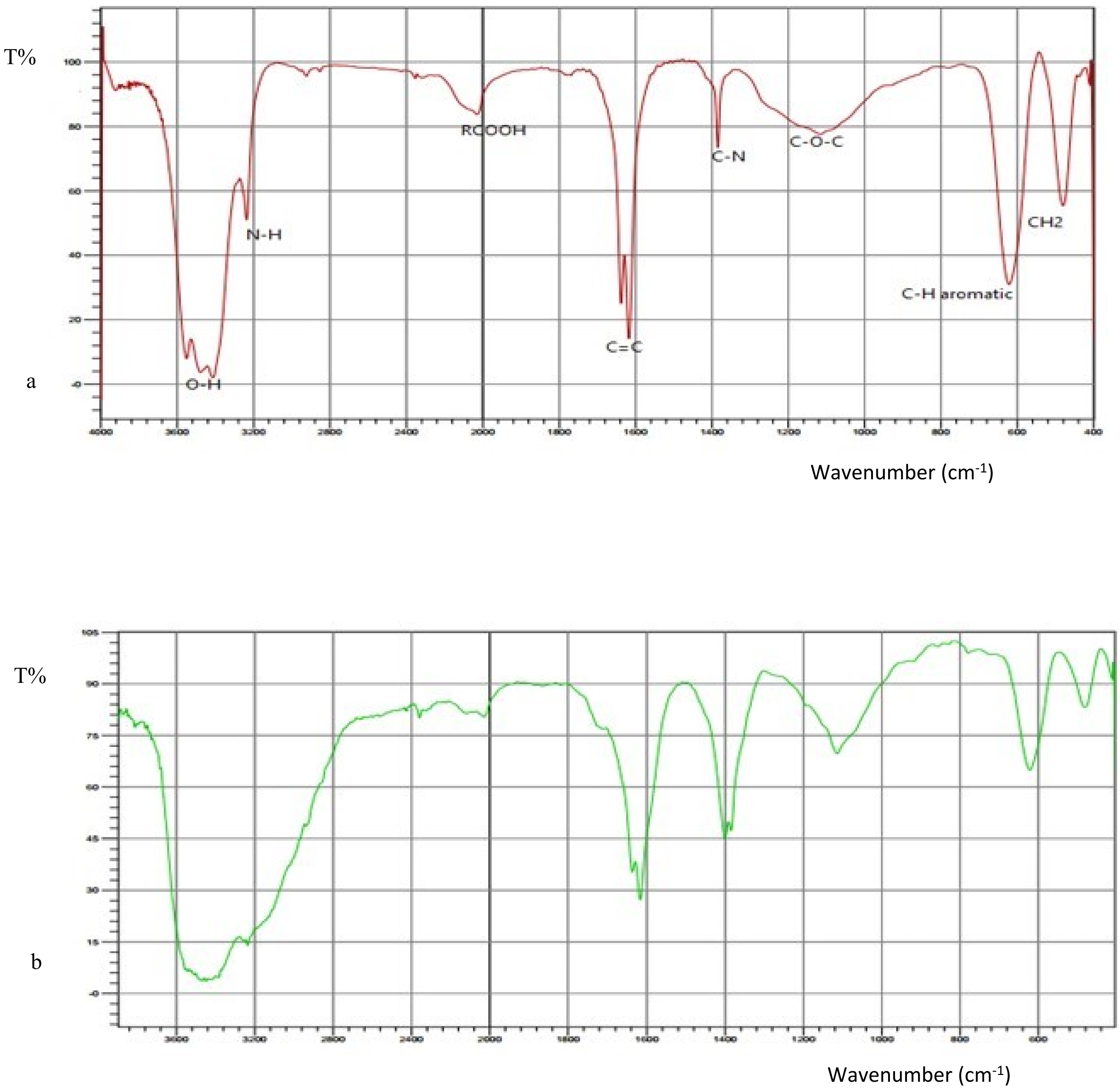
T%
RCOOH
N-H
O-H
C=C
C-N
C-O-C
CH2
C-H aromatic
a
Wavenumber (cm-1)
T%
b
Wavenumber (cm-1)

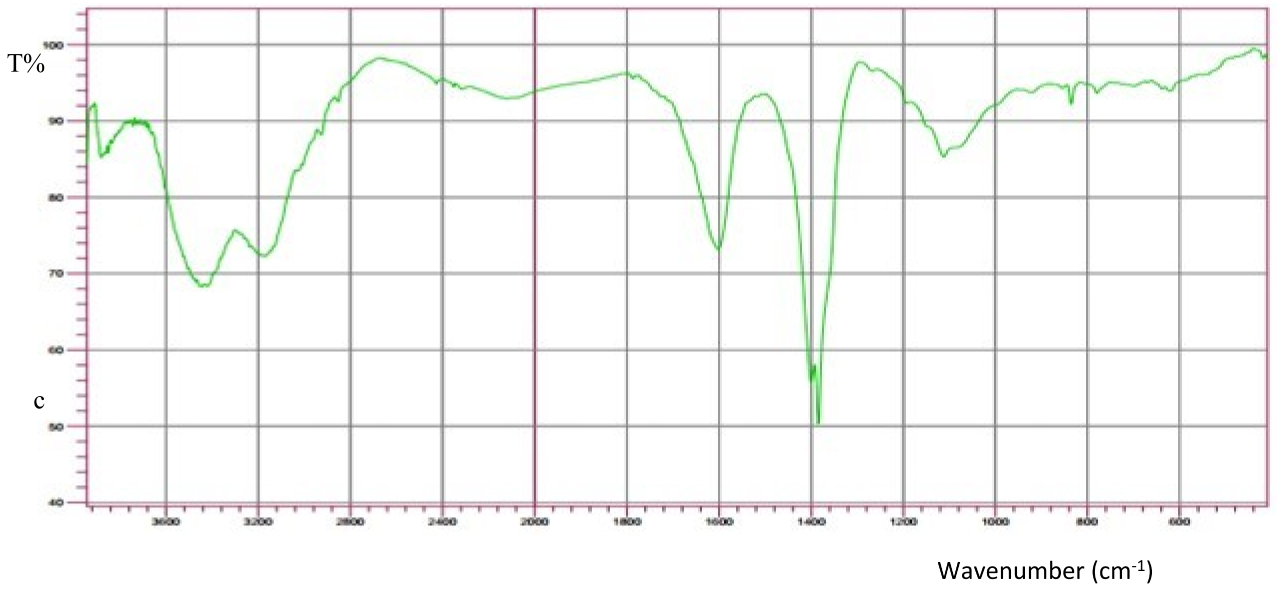

Figure.S.9. a) FT-IR spectra of GQDs (glucose precursor).**b)** FT-IR spectra of Rb-GQDs (Rb concentration is 10 mg .L^{-1}).**c)** FT-IR spectra of Rb-GQD (Rb concentration is 30 (mg.L^{-1})which is the optimum concentration).

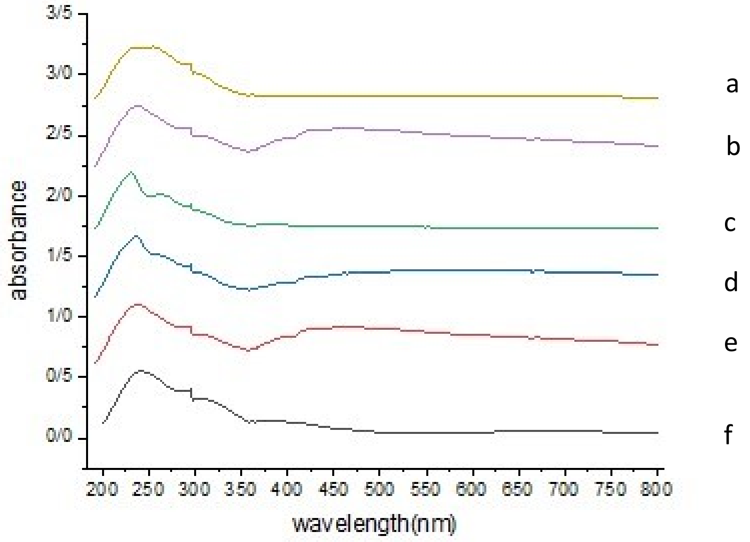

Figure S.10. UV-Vis spectra of a) GQD b)Cu -GQD c) Se-GQD d) Ag-GQD e) Au-GQD f) Rb-GQD

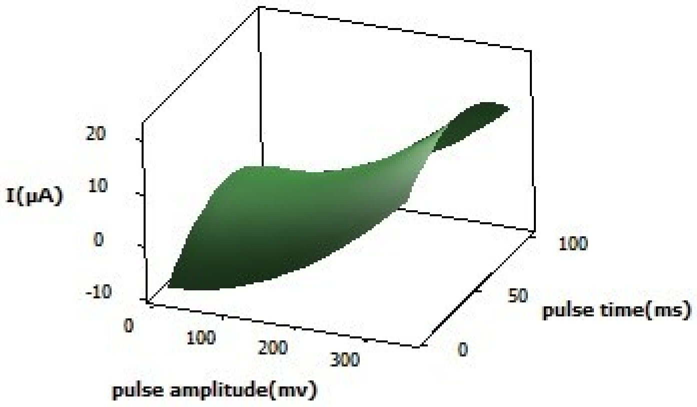

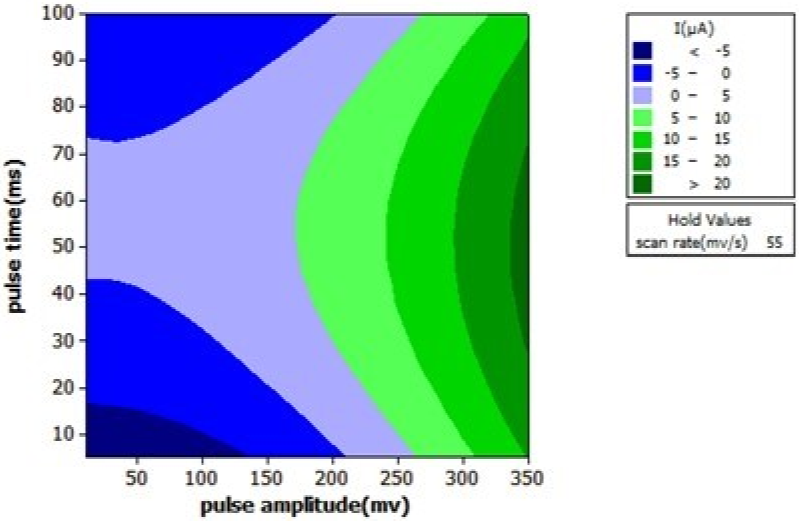

Figure S.11. Surface and contour plot of the interaction effects of DPV analysis parameters.

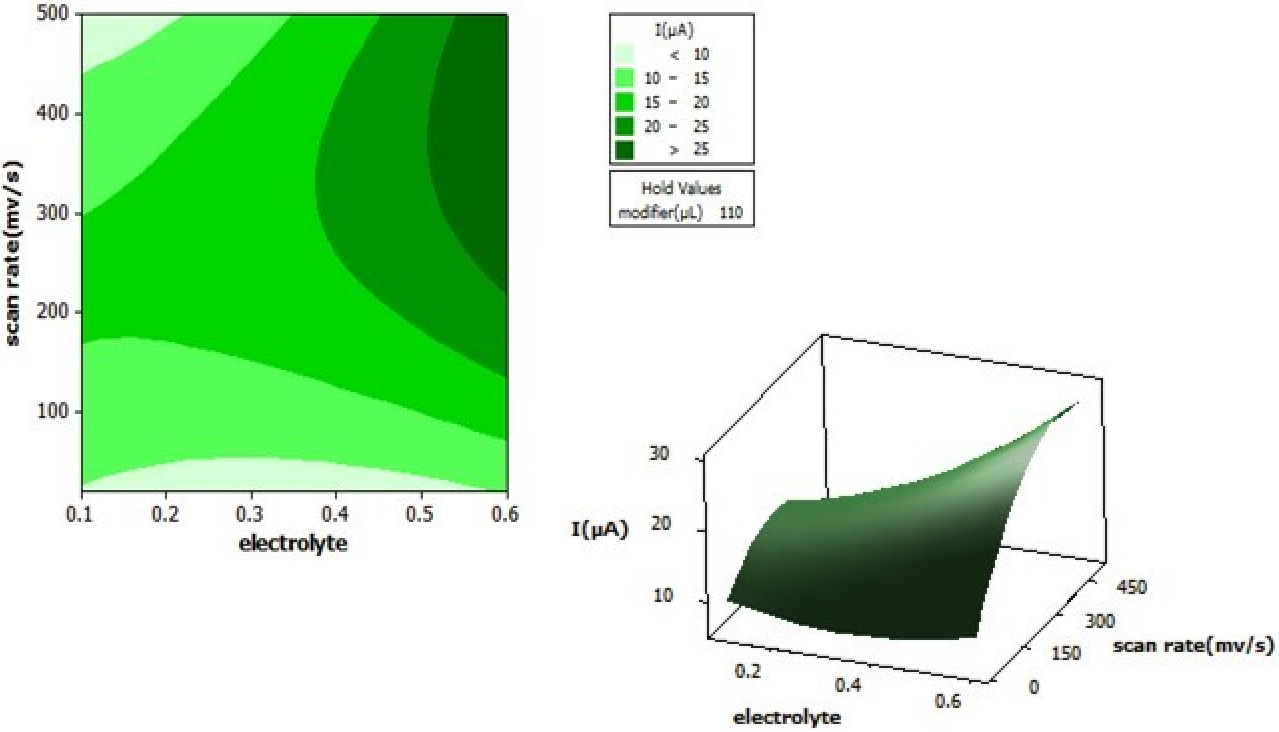

Figure S.12. Plots of optimization of CV parameters and electrolyte concentration.

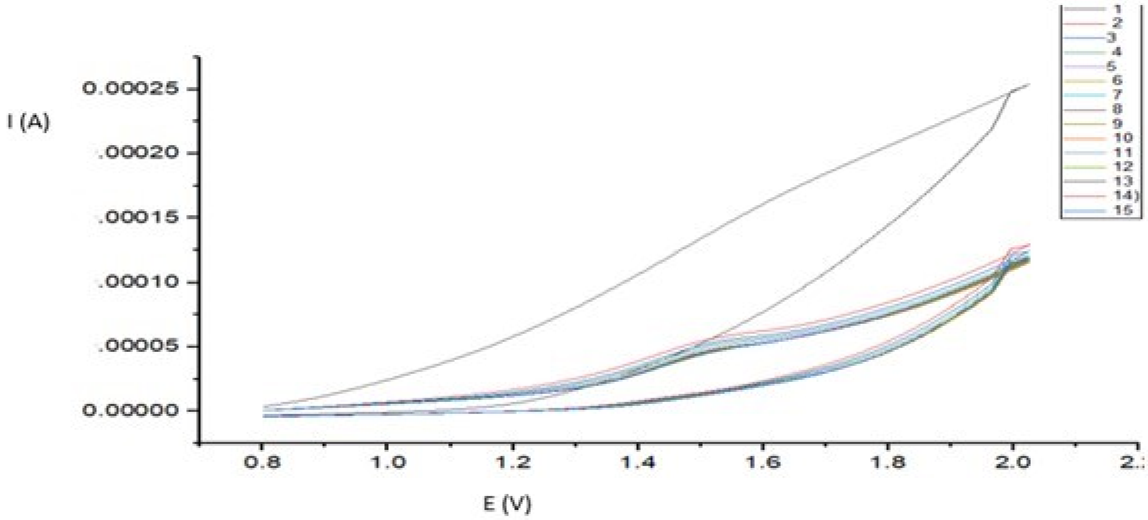

Figure S.13. Consecutive CV voltammograms of DBT

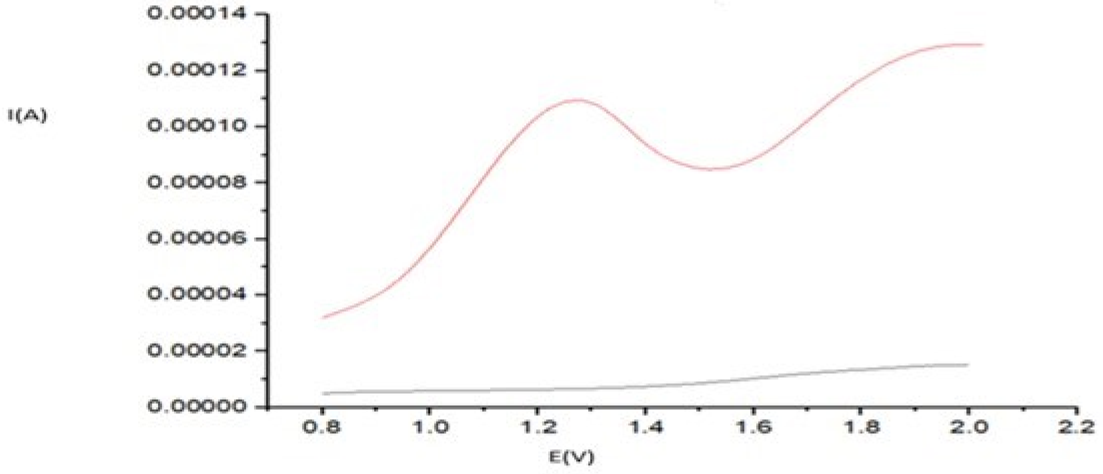

Figure S.14. Current vs. voltage to compare the modified electrode with the bare (unmodified) electrode, here the baselines are not subtracted.

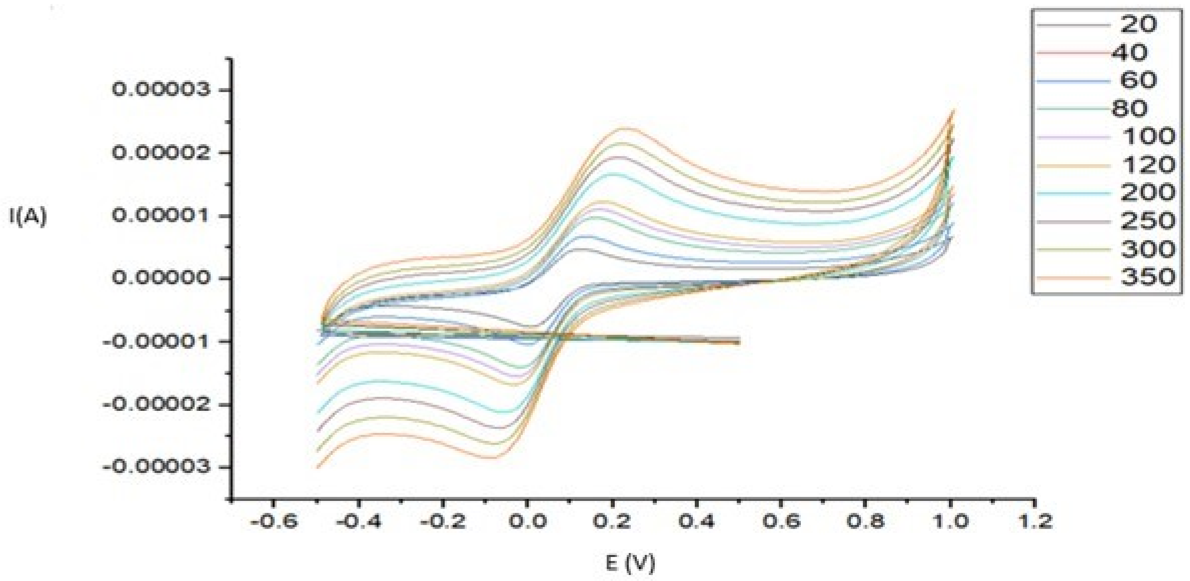

Figure S15. Current curves versus potential related to different scan rates in ferrocyanide prob with bare electrode

Table S1. Experimental design of atomically dispersed Au on the preparation of GQD, C8 is the response column

→	C1	C2	C3	C4	C5	C6	C7	C8
	StdOrder	RunOrder	PtType	Blocks	temprature(°C)	gqd volume (ml)	metal volume(ml)	I (μA)
1	13	1	0	1	100	2	3	1.57
2	10	2	2	1	100	3	1	1.70
3	11	3	2	1	100	1	5	1.81
4	15	4	0	1	100	2	3	1.30
5	4	5	2	1	150	3	3	2.90
6	9	6	2	1	100	1	1	3.79
7	12	7	2	1	100	3	5	3.76
8	8	8	2	1	150	2	5	2.83
9	2	9	2	1	150	1	3	3.21
10	14	10	0	1	100	2	3	1.40
11	7	11	2	1	50	2	5	2.52
12	6	12	2	1	150	2	1	2.60
13	3	13	2	1	50	3	3	4.00
14	1	14	2	1	50	1	3	3.09
15	5	15	2	1	50	2	1	3.36

Table S2. Experimental design of factors of the DPV determinations, C8 is the answer column.

→	C1	C2	C3	C4	C5	C6	C7	C8
	StdOrder	RunOrder	PtType	Blocks	pulse amplitude(mv)	pulse time(ms)	scan rate(mv/s)	I(μA)
1	6	1	2	1	350	52.5	10	59.800
2	2	2	2	1	350	5.0	55	9.870
3	15	3	0	1	180	52.5	55	7.920
4	13	4	0	1	180	52.5	55	5.940
5	8	5	2	1	350	52.5	100	10.300
6	9	6	2	1	180	5.0	10	4.850
7	14	7	0	1	180	52.5	55	2.500
8	4	8	2	1	350	100.0	55	4.600
9	11	9	2	1	180	5.0	100	0.060
10	1	10	2	1	10	5.0	55	0.070
11	5	11	2	1	10	52.5	10	0.489
12	7	12	2	1	10	52.5	100	0.111
13	12	13	2	1	180	100.0	100	3.740
14	3	14	2	1	10	100.0	55	0.210
15	10	15	2	1	180	100.0	10	10.100

Table S3. The experimental design of factors of the CV determinations, c8 is the answer column.

↓	C1	C2	C3	C4	C5	C6	C7	C8
	StdOrder	RunOrder	PtType	Blocks	electrolyte	scan rate(mv/s)	modifier(μL)	I(μA)
1	12	1	2	1	0.35	500	200	8.77
2	11	2	2	1	0.35	20	200	8.56
3	1	3	2	1	0.10	20	110	8.01
4	15	4	0	1	0.35	260	110	20.00
5	4	5	2	1	0.60	500	110	30.10
6	5	6	2	1	0.10	260	20	9.44
7	8	7	2	1	0.60	260	200	19.70
8	10	8	2	1	0.35	500	20	5.70
9	9	9	2	1	0.35	20	20	1.32
10	14	10	0	1	0.35	260	110	17.70
11	13	11	0	1	0.35	260	110	18.60
12	3	12	2	1	0.10	500	110	9.80
13	7	13	2	1	0.10	260	200	9.10
14	2	14	2	1	0.60	20	110	6.65
15	6	15	2	1	0.60	260	20	25.40
